# Differences in glutamate uptake between cortical regions impact neuronal NMDA receptor activation

**DOI:** 10.1038/s42003-019-0367-9

**Published:** 2019-04-05

**Authors:** Jennifer Romanos, Dietmar Benke, Aiman S. Saab, Hanns Ulrich Zeilhofer, Mirko Santello

**Affiliations:** 1 Institute of Pharmacology and Toxicology, University of Zurich, CH-8057 Zurich, Switzerland; 2 Neuroscience Center Zurich, University of Zurich and ETH Zurich, CH-8057 Zurich, Switzerland; 3 Institute of Pharmaceutical Sciences, ETH Zurich, CH-8093 Zurich, Switzerland

## Abstract

Removal of synaptically-released glutamate by astrocytes is necessary to spatially and temporally limit neuronal activation. Recent evidence suggests that astrocytes may have specialized functions in specific circuits, but the extent and significance of such specialization are unclear. By performing direct patch-clamp recordings and two-photon glutamate imaging, we report that in the somatosensory cortex, glutamate uptake by astrocytes is slower during sustained synaptic stimulation when compared to lower stimulation frequencies. Conversely, glutamate uptake capacity is increased in the frontal cortex during higher frequency synaptic stimulation, thereby limiting extracellular buildup of glutamate and NMDA receptor activation in layer 5 pyramidal neurons. This efficient glutamate clearance relies on Na^+^/K^+^-ATPase function and both GLT-1 and non-GLT-1 transporters. Thus, by enhancing their glutamate uptake capacity, astrocytes in the frontal cortex may prevent excessive neuronal excitation during intense synaptic activity. These results may explain why diseases associated with network hyperexcitability differentially affect individual brain areas.

## Abstract (web summary)

Jennifer Romanos et al. investigate how differences in astrocyte-mediated glutamate clearance between the frontal and somatosensory cortex impact neuronal function. They find that the frontal cortex more efficiently clears glutamate when synaptic stimulation frequency is high and this may prevent excessive neuronal excitation during sustained synaptic activity.

## Introduction

Glutamate clearance from the extracellular space during neuronal activity is crucial to spatially and temporally limit neuronal activation^1^. Impaired glutamate clearance favors a number of neurological disorders including migraine and epilepsy. Upon neuronal excitation, glutamate is released into the synaptic cleft with a very fast and sharp time course reaching 1 mM for only 1–2 ms, which quickly returns to nanomolar levels^2–4^. If glutamate were to persist in the extracellular space at high concentrations, it would tonically activate glutamatergic receptors on nearby spines and dendrites, thus compromising the temporal and spatial specificity of synaptic transmission and increasing neuronal activity^5^. It is therefore of vital importance that glutamate is rapidly and efficiently cleared from the extracellular space. In the adult brain, very efficient glutamate transporters (GluTs) are the predominant way to clear glutamate^6^. GluTs are expressed on both neurons and astrocytes. However, following neuronal activity most glutamate uptake is via astrocytic GluTs facing the synapse^7,8^. Glutamate uptake has been thought to be invariable and independent of neuronal activity because of the high expression of GluTs on astrocytes. However, recent evidence shows that glutamate uptake by astrocytes is subject to activity-dependent modulation^9–11^. Thus, during sustained presynaptic activity in the adult somatosensory cortex, the glutamate uptake capacity of astrocytes gradually decreases, likely leading to increased glutamate in the extracellular space^10,12^. Yet, whether this is a general principle in the adult cortex is not clear. The somatosensory cortex and the frontal cortex are cortical regions that serve distinct functions (sensory versus associational/motivational/attentional) and whose neurons also feature different properties^13^. Neuronal activity patterns in these two brain areas are also quite different (sparse firing versus recurrent firing), and region-specific molecular mechanisms may be required to cope with such distinctive network activity regimes. We therefore examined possible differences in glutamate uptake dynamics in these two brain regions by studying glutamate clearance in cortical layer 1, which receives both local and long-range axons from different brain areas. The spatiotemporal convergence of the different inputs in this layer strongly impacts how neurons integrate incoming synaptic stimuli. Defining how the glutamate uptake system influences glutamate spillover and the activation of neuronal receptors can therefore shed light on how long-range and local inputs are integrated within a single neuron. Using a combination of patch-clamp recordings from astrocytes, neurons and dendrites, two-photon glutamate imaging, western blotting and q-RT PCR, we show here that in the frontal cortex glutamate uptake capacity increases upon sustained neuronal activity, thereby preventing runaway synaptic excitation.

## Results

### Different glutamate uptake kinetics between cortical areas

**Fig. 1:**
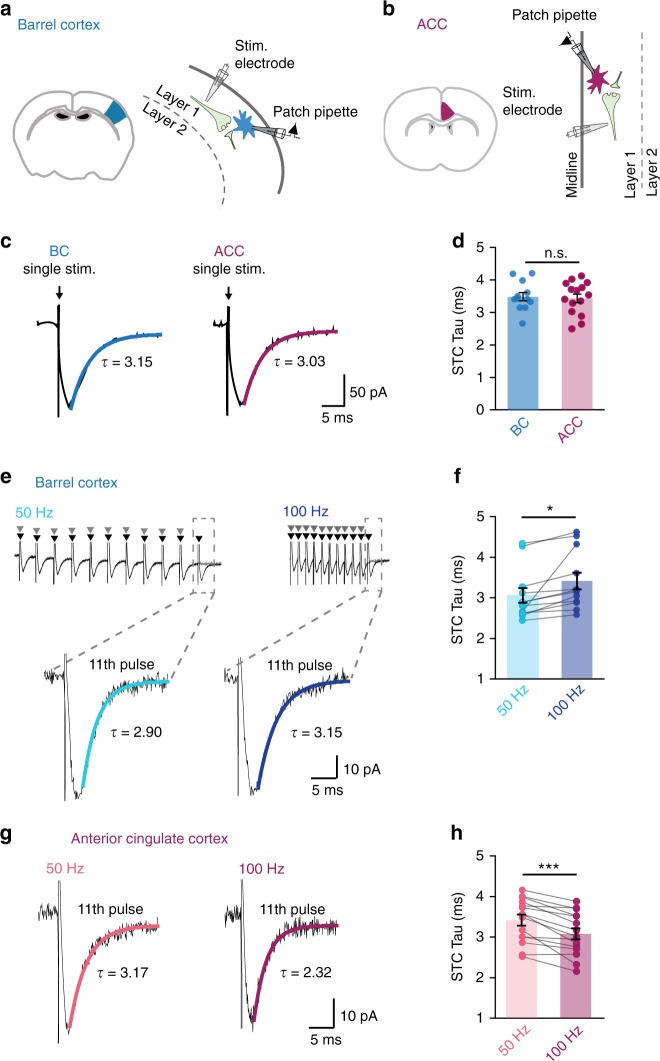
Astrocytic glutamate uptake is facilitated in the ACC during high-frequency synaptic stimulation. **a**, **b** Schemes representing the astrocytic whole-cell patch-clamp recordings in layer 1 of the barrel cortex (**a**) and in layer 1 of the ACC (**b**) in acute brain slices. Currents were evoked by focal electrical stimulation via a theta-glass pipette placed in layer 1 in proximity to the recorded astrocyte. **c** Representative traces of the inward current evoked in an astrocyte by a single pulse stimulation in the barrel cortex (left) and in the ACC (right). **d** The average decay time of the STCs elicited by a single stimulation in the barrel cortex (*τ*_decay_ = 3.5 ± 0.13 ms; *n* = 12) is similar to that in the ACC (*τ*_decay_ = 3.45 ± 0.13 ms; *n* = 15). **e** Superimposed representative traces of the inward current evoked in an astrocyte by an extracellular stimulation train of 11 pulses (black) and a train of 10 pulses (gray) at 50 Hz and 100 Hz in the barrel cortex. **f** In the barrel cortex, the average STCs decay time elicited by the 11th pulse of 50 Hz trains is significantly faster than that elicited by the 11th pulse of 100 Hz trains (*n* = 12, *N* = 7, *P = 0.0162) each point represents the STC decay time in one astrocyte at 50 Hz (light blue) then at 100 Hz (dark blue). **g** Same as (**e**) for the anterior cingulate cortex. **h** In the ACC, the average STCs decay time elicited by the 11th pulse of 50 Hz trains is significantly slower than that elicited by the 11th pulse of 100 Hz trains (*n* = 15, *N* = 6, ***P < 0.0001). Each point represents the STC decay time of one astrocyte at 50 Hz (pink) then at 100 Hz (purple). Representative traces are the average of at least five sweeps. *n* = number of cells, *N* = number of mice. Data are mean ± SEM. Two-tailed paired *t* test

To compare glutamate clearance by astrocytes in different cortical regions, we recorded synaptically activated glutamate transporter currents (STCs) from individual astrocytes in adult acute brain slices. Whole-cell patch-clamp recordings were performed from astrocytes in layer 1 of the somatosensory cortex (barrel, BC Fig. 1a) and in layer 1 of the frontal cortex (anterior cingulate, ACC, Fig. 1b) in the presence of AMPA, NMDA, and GABA_A_ receptor antagonists (CNQX 10 μM, AP-5 50 μM and Picrotoxin 100 μM) while synapses were focally stimulated in the proximity of the recorded astrocyte (Fig. 1a, b). The decay kinetics of the STCs provides a good estimate of how rapidly synaptically released glutamate is taken up by astrocytes^14^ (but see Discussion). We fitted a mono-exponential curve to the STC decay and calculated the time constant tau (*τ*). STCs decay kinetics were similar in the BC (*τ*_decay_ = 3.5 ± 0.13 ms, *n* = 12 cells) and in the ACC (*τ*_decay_ = 3.45 ± 0.13 ms, *n* = 15) following a single stimulation (Fig. 1c, d), suggesting that upon temporally sparse neuronal activity, glutamate uptake is comparable between the two cortical regions. We subsequently sought to compare STC kinetics between the two cortical regions during high-frequency synaptic activity. We evoked STCs by trains of 10 and 11 pulses at 50 and 100 Hz. We analyzed the decay kinetics of the last pulse by subtracting the current elicited by 10 pulses from that elicited by the 11th pulse (Fig. 1e–g) similar to^10^. Consistent with previous reports^12^, decay kinetics of STCs evoked at 100 Hz (Fig. 1f) were slower than those evoked at 50 Hz (50 Hz: *τ*_decay_ = 3.01 ± 0.19 ms, 100 Hz: *τ*_decay_ = 3.36 ± 0.21 ms) in the barrel cortex (*n* = 12, *P = 0.016). Hence, as expected, glutamate uptake by astrocytes in the somatosensory cortex slows down during high-frequency synaptic stimulation (100 Hz). Surprisingly, in the ACC STC decay becomes significantly faster following 100 Hz stimulation (Fig. 1h) compared with 50 Hz stimulation (100 Hz: *τ*_decay_ = 3.08 ± 0.01 ms, 50 Hz: *τ*_decay_ = 3.42 ± 0.14 ms; *n* = 15, ***P < 0.0001). These data suggest that astrocytes in different cortical regions, albeit situated in the same cortical layer, display opposite short-term plasticity of STC kinetics. To check for any other possible region-specific differences between the two astrocytic populations, we compared their morphological and electrophysiological properties (Supplementary Fig. 1). Besides having a smaller cell body size (Supplementary Fig. 1a–b), which parallels the smaller neuronal soma size, the number of astrocytes in the ACC, their input resistance and their resting membrane potential were comparable to those located in the barrel cortex (Supplementary Fig. 1c–e). Hence the difference in STC short-term plasticity of ACC astrocytes is not accompanied by variations in other gross morphological or electrophysiological properties.

### Increased glutamate uptake capacity in the frontal cortex

**Fig. 2:**
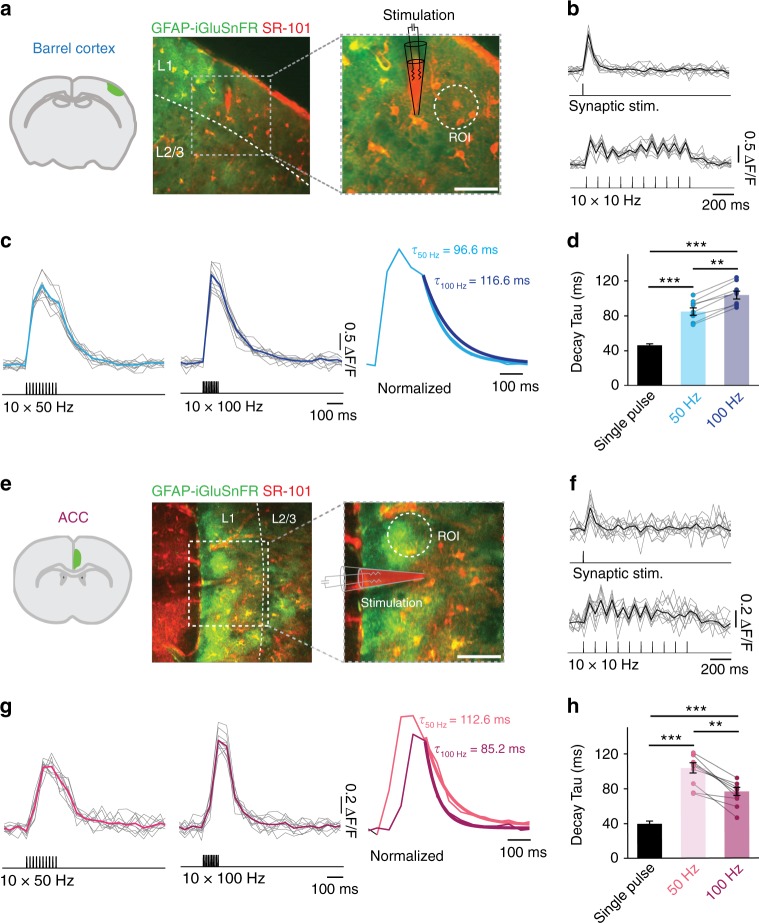
Extrasynaptic glutamate is cleared faster during high-frequency stimulations in the ACC in contrast to the BC. **a** Typical experiment showing the extent of expression of iGluSnFR in the adult mouse barrel cortex. The glutamate sensor was expressed specifically on the plasma membrane of cortical astrocytes (GFAP-iGluSnFr, green channel). In red, astrocytes were stained with Sulforhodamine-101 (SR-101). Theta-glass electrode for synaptic stimulation was placed in the inner layer one and glutamate was imaged from a region of interest (ROI) adjacent to the electrode. Scale bar = 40 μm. **a**–**c** Upon synaptic stimulation: single stimulation, 10 × 10 Hz (**b**), 10 × 50 Hz, and 10 × 100 Hz (**c**) robust and consistent increases in iGluSnFr emission could be detected. Thick lines represent the average of the responses and the mono-exponential fit of the decay. **d** The decay kinetics of the averaged transients are slower following 100 Hz stimulation compared with 50 Hz (*n* = 8, **P < 0.001) and decay kinetics of the transients at both 50 and 100 Hz are significantly slower than those from a single pulse (*n* = 8, *N* = 3, ***P < 0.0001). **e**–**g** same as (**a**–**c**) for the ACC. Scale bar = 40 μm. **h** The decay kinetics of the averaged transients are faster following 100 Hz stimulations compared with 50 Hz (*n* = 8, **P < 0.001) and decay kinetics of the transients at both 50 and 100 Hz are significantly slower than those at single pulse (*n* = 8, *N* = 3, ***P < 0.0001). *n* = number of slices, *N* = number of mice. Data are mean ± SEM. One-way ANOVA with Bonferroni post hoc test

STCs represent an indirect measure of extrasynaptic glutamate dynamics, inevitably distorted by the electrical filtering properties of the astrocyte and possibly influenced by other synaptically activated conductances. As an alternative approach to measure extrasynaptic glutamate in the BC and ACC, we took advantage of the intensity-based glutamate fluorescent sensor iGluSnFr^15^ that we specifically expressed on the extracellular side of the astrocytic plasma membrane (see Methods). iGluSnFr sensor is suitable to measure relative variations in extracellular glutamate lifetime both in vitro and in vivo^16,17^ and has been proven to be sensitive to minor changes in GluT activity^10,18,19^ (see Supplementary Discussion on the significance of iGluSnFr measures). We therefore employed iGluSnFr in combination with two-photon microscopy and studied the time course of extrasynaptic glutamate with high spatial and temporal resolution following synaptic stimulation. Synaptic activity was evoked by focal electrical stimulation in cortical layer 1 in the BC and ACC and glutamate was imaged in a region 10–40 μm away from the stimulation electrode (Fig. 2a, e). The signal appeared to be robust and reproducible upon several repetitive trials (Fig. 2b, c, f, g) and was not affected by Sulforhodamine 101 (SR-101) employed for astrocyte visualization (Supplementary Fig. 2). To estimate the speed of glutamate clearance, we fitted the decay of the averaged evoked glutamate transients (ten trials) with a mono-exponential curve^18^. Upon single stimulation or temporally sparse synaptic activity (ten pulses at 10 Hz) glutamate decayed with similar kinetics in both cortical regions (Fig. 2b–f; single stimulation: in BC *τ*_decay_ = 44.78 ± 2.48 ms, in ACC *τ*_decay_ = 41.55 ± 1.94 ms; BC: *n* = 18 slices, ACC: *n* = 14 slices, *P* = 0.33. 10 Hz stimulations: in BC *τ*_decay_ = 60.74 ± 4.9 ms, in ACC *τ*_decay_ = 50.14 ± 4.9 ms; BC: *n* = 16 slices, ACC: *n* = 15 slices, *P* = 0.14). Consistent with previous findings^10,18^, stimulations at 50 Hz led to a substantially slower decay of glutamate transients in both cortical regions (Fig. 2c, d, g, h). In the barrel cortex, increasing the stimulation frequency to 100 Hz (Fig. 2c, d) further slowed the decay of the glutamate signals (50 Hz: *τ*_decay_ = 84.79 ± 4.28 ms; 100 Hz: *τ*_decay_ = 103.86 ± 4.58 ms; *n* = 8 slices, **P < 0.001). This is consistent with the notion that GluTs in this area may display reduced glutamate uptake capacity during high-frequency stimulations and is consistent with the slowdown of STCs decays recorded from astrocytes. Conversely, when we compared 50 Hz and 100 Hz stimulation in the ACC, the time course of extrasynaptic glutamate appeared drastically different: glutamate was cleared faster from the extracellular space at 100 Hz compared with 50 Hz (50 Hz: *τ*_decay_ = 103.75 ± 5.86 ms, 100 Hz: *τ*_decay_ = 76.63 ± 4.65 ms, *n* = 9 slices, **P < 0.001) as indicated by a ~21% smaller time constant (Fig. 2g, h). Remarkably, the decay of glutamate transients did not correlate with the amplitude of the synaptically evoked iGluSnFr signals at a given stimulation paradigm (Supplementary Fig. 3), demonstrating that, within an individual brain region, the decay of the iGluSnFr transients is not influenced by the amplitude of the response but rather by the frequency of the stimuli (Fig. 2 and Supplementary Fig. 3). Consistently, the decay kinetics of iGluSnFR responses were independent of whether the region of interest was placed close to the tip of the stimulation electrode (where the response was nearly maximal) or more distally (Supplementary Fig. 3). Moreover, the facilitation of glutamate uptake did not depend on differences in synaptic glutamate release, as the synaptic responses recorded from layer 5 pyramidal neurons were comparable between the two regions upon single stimulations (AMPA currents in BC: −105.4 ± 14.5 pA, *n* = 9; in ACC: −97.7 ± 19.9 pA; *n* = 8, *P* = 0.75) and short-term synaptic plasticity of both AMPA and NMDA currents was also similar (Supplementary Fig. 4a–d). Similar synaptic properties between the two cortical regions were also confirmed by a comparable increase in the amplitude of both iGluSnFr responses and NMDA currents between 50 and 100 Hz synaptic stimulations (Supplementary Fig. 5). Together, our data indicate that in the ACC, contrary to the barrel cortex, glutamate uptake increases with higher frequency synaptic stimulation.

**Fig. 3:**
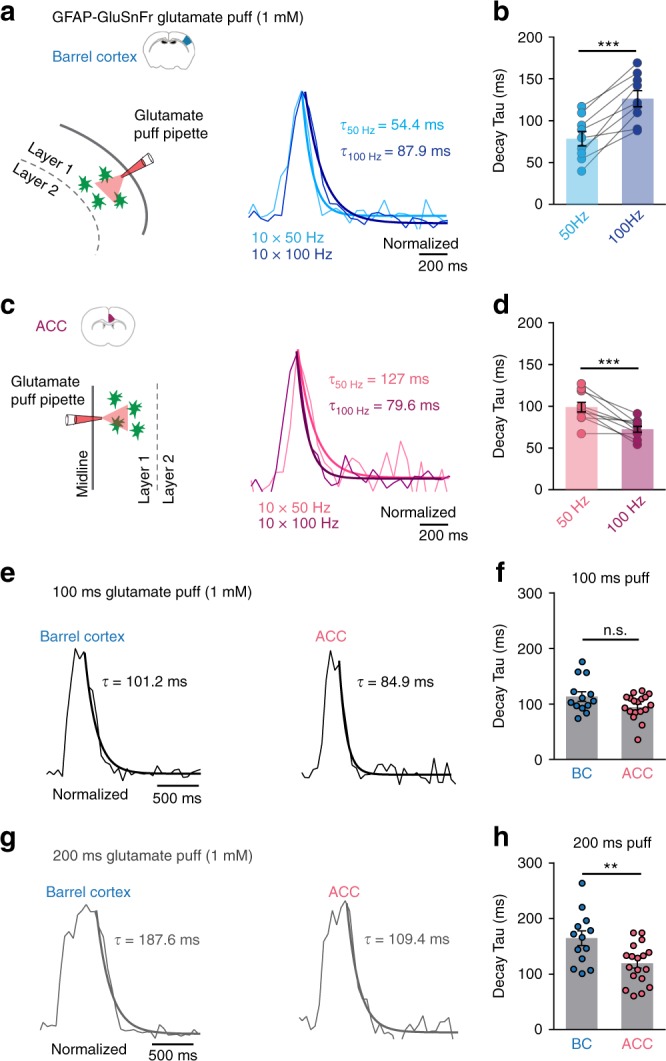
Exogenous glutamate is scavenged differently in the BC and in the ACC. **a** Left: cartoon depicting localized glutamate puff (1 mM) through a pipette in layer 1 of the barrel cortex. Right: example traces of the glutamate puff-evoked iGluSnFr responses triggered by ten short (8 ms) puffs at 50 Hz (light blue) and 100 Hz (dark blue) in the BC. **b** The decay of the glutamate puff-evoked iGluSnFr responses in the BC upon 100 Hz stimulation is slower compared with 50 Hz (*n* = 9, ***P = 0.0001). **c** Left: same as **a** but in the ACC. Right: Example traces of the glutamate puff-evoked iGluSnFr responses triggered by ten short puffs at 50 Hz (light pink) and 100 Hz (dark pink) in the ACC. **d** The decay of the glutamate puff-evoked iGluSnFr responses in the ACC upon 100 Hz stimulation is faster compared with 50 Hz (*n* = 10, ***P = 0.0009). **e** Example traces of glutamate puff-evoked iGluSnFr responses triggered by continuous 100 ms in the BC and in the ACC. **f** The decay of glutamate puff-evoked iGluSnFr responses by 100 ms stimulations was slightly but not significantly slower in the BC compared with the ACC (BC: *n* = 13, ACC: *n* = 18, *P* = 0.059). **g** Example traces of 200 ms glutamate puffs in the BC and in the ACC. **h** Upon continuous 200 ms glutamate puffs, the decay of iGluSnFr evoked signals were significantly slower in the BC compared with the ACC (BC: *n* = 13, *N*_BC_ = 3, ACC: *n* = 18, *N*_ACC_ = 3, **P = 0.0053). Traces normalized to the peak. *n* = number of slices, *N* = number of mice. Data are mean ± SEM. Two-tailed paired *t* test and two-tailed unpaired *t* test

To assess how exogenous glutamate, i.e., of non-synaptic origin, is cleared in the two cortical regions, we applied localized glutamate puffs and imaged glutamate-evoked iGluSnFR signals in layer 1 of both cortical regions (Fig. 3a–c). We employed an externally triggered pressure ejector to apply trains of ten short (8 ms) puffs at 50 and 100 Hz or continuous puffs of 100 and 200 ms. Consistent with our synaptic stimulation experiments, the decay of the glutamate puff-evoked iGluSnFr responses upon 100 Hz stimulation was slower compared with 50 Hz in the BC (Fig. 3b, 50 Hz: 78.9 ± 8.6 ms, 100 Hz: 126.8 ± 9.6 ms, *n* = 9, ***P = 0.0001). Conversely, in the ACC, the decay time constant of the iGluSnFr responses was faster for 100 Hz stimulation compared with 50 Hz (Fig. 3d, 50 Hz: 99.2 ± 5.8 ms, 100 Hz: 72.7 ± 3.5 ms, *n* = 10, ***P = 0.0009). In addition, we examined how exogenous glutamate is scavenged when it is continuously applied for prolonged durations (100 and 200 ms) in both cortical regions. We found that upon continuous 200 ms glutamate puffs (Fig. 3g, h), the decay of iGluSnFr signals were significantly slower in the BC compared with ACC (BC: 164.7 ± 13.2 ms, *n* = 13; ACC: 119.5 ± 8.5 ms, *n* = 18, **P = 0.0053). No significant difference was observed (albeit a trend was detected) for 100 ms puff applications (Fig. 3e, f, in BC: 113.2 ± 8.6 ms, *n* = 13; in ACC: 94.5 ± 5.3 ms, *n* = 18; *P* = 0.059). Importantly, the glutamate-evoked iGluSnFr decay-time constants were strongly reduced by sub-saturating concentrations of the GluT blocker DL-TBOA, showing that the kinetics of iGluSnFr signal evoked by exogenous glutamate applications is influenced by active glutamate uptake (Supplementary Fig. 6b–c). In summary, our results show that both synaptically released and exogenous glutamate are differentially cleared in the two cortical regions and this difference depends on both the duration of stimulation and on the stimulation pattern.

### Glutamate uptake facilitation in the ACC limits NMDA currents

**Fig. 4:**
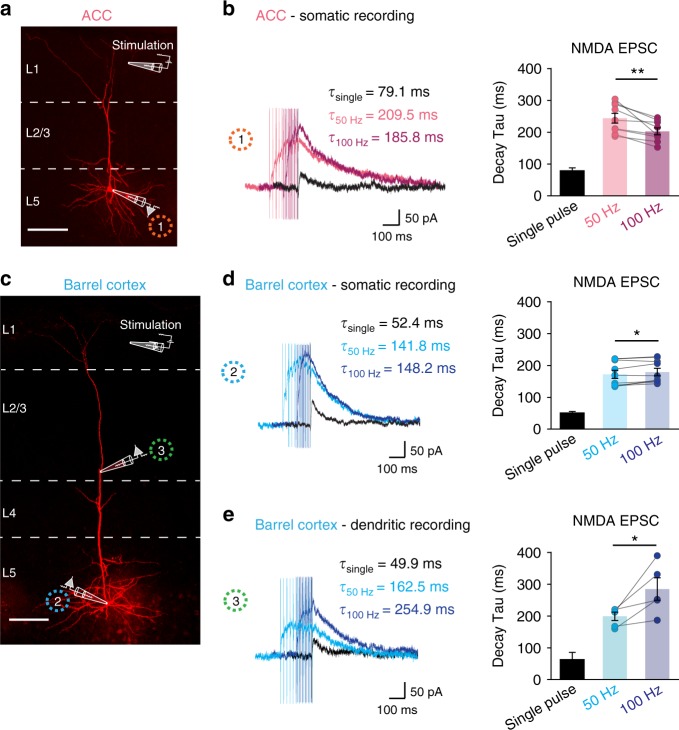
NMDA EPSCs in layer 5 pyramidal neurons are faster following high-frequency stimulations in the ACC. **a** Image of patch-clamp recording from soma of a biocytin-labeled layer 5 (L5) pyramidal cell in the ACC showing the location of the recording and stimulation pipettes (layer 5 and inner layer 1, respectively). Scale bar = 100 μm. **b** Representative traces of NMDA-evoked EPSCs in soma of L5 pyramidal cells in the ACC following single stimulation, trains of 10 stimuli at 50 and 100 Hz (holding potential at + 40 mV). The decay kinetics of NMDA-evoked EPSCs are faster following trains of 100 Hz stimulation than following 50 Hz stimulation (*n* = 9, *N* = 6, **P = 0.005). **c** Image of a biocytin-labeled L5 pyramidal cell in the BC. Whole-cell patch-clamp recordings were either acquired from the soma or from the apical dendrite. Scale bar = 100 μm. **d** Somatic recordings: representative traces of NMDA-evoked EPSCs in soma of L5 pyramidal cells in the BC following single stimulation, trains of 10 stimuli at 50 and 100 Hz. The decay kinetics of NMDA-evoked EPSCs in the soma of L5 pyramidal cells is slightly slower (by 4%) upon 100 Hz stimulations compared with 50 Hz stimulations (*n* = 9, *N* = 6, *P = 0.025). **e** Dendritic recordings: representative traces of NMDA-evoked EPSCs in the recorded dendrites. The decay kinetics of NMDA-evoked EPSCs in the dendrites of L5 pyramidal cells becomes 43.2% slower upon 100 Hz stimulations compared with 50 Hz stimulations (*n* = 5, *N* = 5, *P = 0.04). *n* = number of cells, *N* = number of mice. Data are mean ± SEM. Two-tailed paired *t* test

We then wondered which consequences the facilitation of glutamate buffering had on neuronal function. It is well-established that changes in glutamate uptake primarily affect the activation of NMDA receptors, which possess high affinity for glutamate^10,20^. We therefore measured synaptic NMDA currents from somata of layer 5 pyramidal neurons in the ACC evoked by different synaptic stimulation patterns applied in layer 1 (Fig. 4a, stimulation of synapses in the proximal tuft dendrites). Currents were recorded at + 40 mV in presence of the NMDAR co-agonist D-serine and AMPA/GABA_A_ receptor antagonists. We compared NMDA currents evoked by 50 and 100 Hz synaptic stimulation (Fig. 4b) and found different decay kinetics: the decay was consistently faster at 100 Hz compared with 50 Hz (50 Hz: *τ*_decay_ = 243.66 ± 15.18 ms, 100 Hz: *τ*_decay_ = 202.41 ± 10.65 ms; *n* = 9 cells, **P = 0.005) as expected by a more efficient clearance of glutamate at higher synaptic stimulation frequencies. When repeating similar recordings from layer 5 pyramidal cells in the BC (Fig. 4c, d), the decay of NMDA currents was always slightly but significantly slower at 100 Hz compared with 50 Hz stimulation (50 Hz: *τ*_decay_ = 172.04 ± 12.72 ms, 100 Hz: *τ*_decay_ = 179.52 ± 11.86 ms; *n* = 9 cells, *P = 0.025). This is in line with our functional data showing increased glutamate lifetime upon higher frequency synaptic activity in this cortical area (Fig. 2). Furthermore, the change in decay kinetics of NMDA currents at 50 and 100 Hz was notably smaller compared with the striking difference of iGluSnFr transients evoked by the same synaptic stimulation patterns in the BC. We hypothesized that this smaller degree of slowing down of NMDA currents (only 4.3%) might be caused by the intensive dendritic filtering properties of the long apical dendrite of layer 5 (L5) pyramidal cells of the BC^21,22^. In fact, these dendrites progressively slow down the time course of synaptic potentials as they spread from the dendritic site of generation to the soma, and this slowdown is more pronounced for synaptic currents with faster initial kinetics^23–25^. This filtering is instead reduced in the shorter apical dendrites of the ACC (Fig. 4), thus allowing a more faithful transmission of distal synaptic inputs to the soma^13^. We therefore decided to measure synaptic NMDA currents directly from apical dendrites of L5 pyramidal cells in the BC as distally as possible from the soma (Fig. 4c, e). Indeed, recordings from apical dendrites revealed that trains of 100 Hz stimulation induced strikingly slower NMDA currents by about 43% compared with 50 Hz stimulation (Fig. 4e, 50 Hz: *τ*_decay_ = 198.9 ± 13.4 ms, 100 Hz: *τ*_decay_ = 284.7 ± 35.3 ms; *n* = 5 cells, *P = 0.04). These data are in agreement with the notion that in the BC, in contrast to the ACC, glutamate uptake is reduced upon higher frequency synaptic activity (50 Hz versus 100 Hz) and suggest that these differences may particularly impact pyramidal cell dendritic excitability.

### Role of K^+^ uptake and Na^+^/K^+^-ATPase in glutamate clearance

**Fig. 5:**
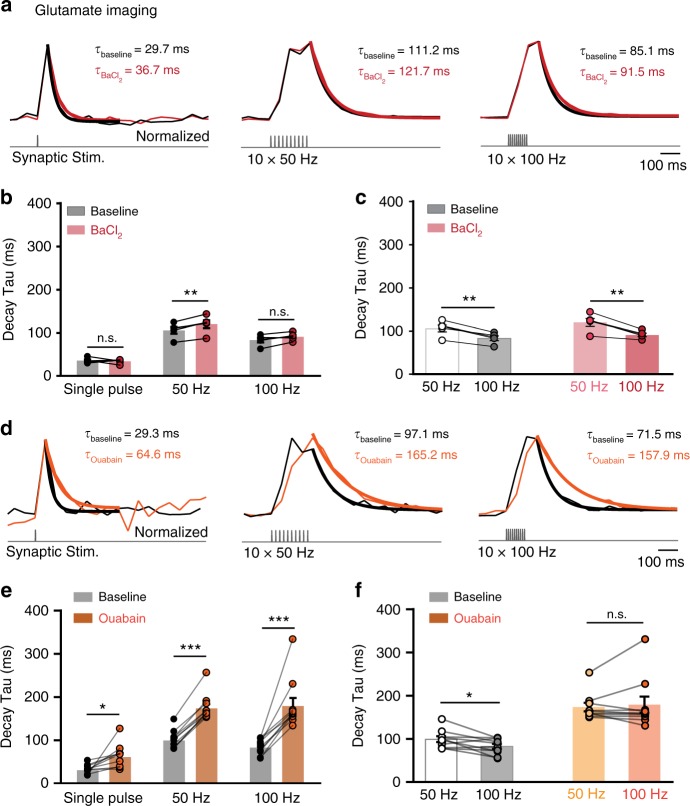
Role of K^+^ uptake and Na^+^/K^+^-ATPase activity in glutamate clearance in the ACC. **a** Transients of iGluSnFR signals at baseline (black traces) evoked by single stimulation, trains of 50 Hz and 100 Hz stimuli and in the presence of 200 µM BaCl_2_ (red traces). **b** Blockade of KIR-dependent K^+^ uptake by BaCl_2_ did not affect glutamate decay kinetics following a single stimulation (baseline: 35.6 ± 2.7 ms; BaCl_2_: 34.3 ± 2.3 ms; *n* = 5, *P* > 0.05). However, BaCl_2_ significantly slowed the decay of iGluSnFR signals in the ACC at 50 Hz by 13% (baseline: 105.9 ± 7.7 ms; BaCl_2_: 119 ± 9.1 ms; *n* = 5 slices, ** *P* < 0.01). The presence of BaCl_2_ had smaller effects on the decay kinetics at 100 Hz (baseline: 83.1 ± 0.1 ms; BaCl_2_: 91.0 ± 8.9 ms, *n* = 5, *P* > 0.05). **c** In the presence of BaCl_2_ in the ACC, the decay of glutamate transients remain significantly faster at 100 Hz (91 ± 8.9 ms) compared with 50 Hz (119 ± 9.1 ms; *n* = 5 slices, *N* = 1, **P = 0.006). Traces normalized to the peak. **d** Same as (**a**), but in the presence of 5 µM ouabain (orange traces). **e** Blockade of Na^+^/K^+^-ATPase by ouabain slowed down glutamate transients drastically at all stimulation intensities with a larger effect at 100 Hz (single pulse, baseline: 30.6 ± 3.2 ms; ouabain: 61.42 ± 8.7 ms; *n* = 10 slices, * *P* < 0.05. At 50 Hz, baseline: 99.3 ± 6.6 ms; ouabain: 173.4 ± 9.7 ms; *n* = 10 slices, ***P < 0.0001. At 100 Hz: baseline: 82.7 ± 5.7 ms; ouabain: 179.1 ± 18.6 ms, *n* = 10, ***P < 0.0001, *n* = number of slices, *N* = number of mice). **f** In the presence of ouabain in the ACC, the decay of glutamate transients becomes comparable, with non-significant difference between 50 and 100 Hz. Traces normalized to the peak. Data are mean ± SEM. Two-way RM ANOVA test and two-tailed paired *t* test

We subsequently sought to investigate the mechanism by which ACC astrocytes improve their glutamate uptake capacity upon high-frequency (100 Hz) synaptic stimulation. We reasoned that increases in intracellular K^+^, which is actively taken up by astrocytes during sustained synaptic activity, could facilitate glutamate uptake. Indeed, since GluTs extrude K^+^ while taking up glutamate^26^, it has been postulated that an increase in cytosolic K^+^ would engender a positive feedback loop that may boost glutamate uptake capacity^27,28^. We therefore bath-applied 200 µM BaCl_2_, which blocks the slow component of the inward K^+^ current evoked in astrocytes by trains of synaptic stimuli^29^. BaCl_2_ only slightly slowed the decay of iGluSnFr-mediated glutamate signals in the ACC upon 50 Hz synaptic stimulation (Fig. 5a, b), but failed to significantly modify the 100 Hz-induced iGluSnFr decay. Because the latter continues to be faster than the 50-Hz-induced glutamate signals (Fig. 5c), we conclude that increases in intracellular K^+^ do not account for the facilitation of glutamate uptake recorded upon high-frequency synaptic activity in the ACC. Na^+^/K^+^-ATPase (NKA) activity has also been reported to play a prominent role in normalizing extracellular K^+^ upon neuronal activity^29,30^ as well as in governing astrocyte Na^+^ homeostasis^31,32^. We therefore sought to pharmacologically block NKA activity with ouabain (5 µM^30^, mostly inhibiting α2- and α3-containing NKA^32^). This approach drastically slowed down the decay kinetics of iGluSnFr signals at all stimulation frequencies (Fig. 5d, e). In the presence of ouabain, the decay kinetics of glutamate transients were comparable between 50 and 100 Hz (Fig. 5f, 50 Hz: 173.4 ± 9.7 ms, 100 Hz: 179.1 ± 18.6 ms; *n* = 10 slices, *P* = 0.57). These results reveal a key role of the NKA in controlling the time course of extracellular glutamate in the ACC, likely by directly regulating the astrocyte electrochemical Na^+^ gradient necessary to maintain efficient glutamate uptake^31,32^ or by influencing activity-mediated intracellular K^+^ rise by astrocytes, which may in turn impact glutamate uptake^29,30,33^.

### GLT-1 contributes to glutamate uptake facilitation in the ACC

**Fig. 6:**
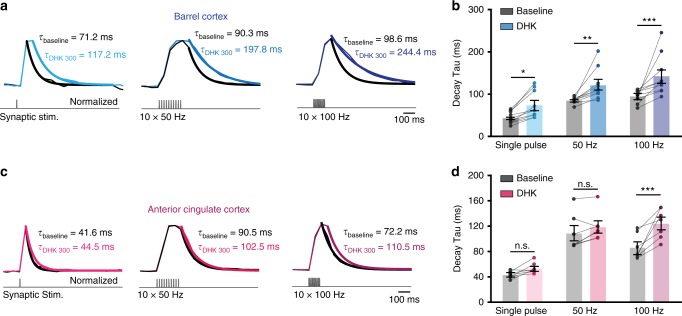
Complete blockade of GLT-1 transporters reverses glutamate uptake facilitation at 100 Hz in the ACC. **a** Representative responses of iGluSnFr signal in the barrel cortex following synaptic stimulation (single stimulation, 10 × 50 Hz and 10 × 100 Hz) during baseline (black traces) and after application of DHK (300 μM, blue traces). **b** Decay kinetics of extrasynaptic glutamate transients are similarly and significantly slower in the presence of DHK (300 μM) following single pulse stimulation (*n* = 10, *P < 0.05), 50 Hz stimulation (*n* = 10, **P < 0.01) and following 100 Hz stimulation (*n* = 10, *N* = 3, **P < 0.001). **c** Same as (**a**) for the ACC. **d** Decay kinetics of extrasynaptic glutamate transients following single pulse and 50 Hz stimulation are not affected by the presence of DHK (*n* = 7, *P* > 0.05). However, following 100 Hz stimulation the decay kinetics become significantly slower in the presence of DHK (*n* = 7, *N* = 3, ***P < 0.0001). Traces normalized to the peak. *n* = number of slices, *N* = number of mice. Data are mean ± SEM. Two-way RM ANOVA test

The two main astrocytic GluTs in the brain are GLT-1 and GLAST. While GLAST mediates most of the glutamate uptake in the cerebellum^34,35^, GLT-1 has been reported to account for the majority of the glutamate uptake in the hippocampus and cortex of the adult brain^35,36^. Consistently, we found that in the barrel cortex, complete and specific blockade of GLT-1 by dihydrokainate (DHK, 300 µM) led to almost doubling of the decay time constant of iGluSnFr glutamate transients, independently of the synaptic activity pattern (Fig. 6a, b; single pulse baseline: *τ*_decay_ = 43.5 ± 4.36 ms; single pulse DHK: *τ*_decay_ = 73.5 ± 9.88 ms; 50 Hz baseline: *τ*_decay_ = 84.62 ± 2.21 ms, 50 Hz DHK: *τ*_decay_ = 120.95 ± 11.43 ms, 100 Hz baseline: *τ*_decay_ = 94.54 ± 4.63 ms, 100 Hz DHK: *τ*_decay_ = 142.23 ± 14.83 ms; *n* = 10; *P_single pulse_ < 0.05, **P_50 Hz_ < 0.01, ***P_100 Hz_ < 0.001). These data confirm that GLT-1 significantly contributes to glutamate uptake in the somatosensory cortex^20^.

Next, we tested the effect of DHK on synaptically evoked glutamate transients in the ACC (Fig. 6c, d). To our surprise, in this brain area blockade of GLT-1 did not alter the temporal dynamics of extrasynaptic glutamate for stimulation frequencies up to 50 Hz (single-pulse baseline *τ*_decay_ = 43.4 ± 2.05 ms, DHK: *τ*_decay_ = 53.56 ± 3.24 ms; 50 Hz baseline: *τ*_decay_ = 108.63 ± 10.67 ms, 50 Hz DHK: *τ*_decay_ = 118 ± 8.37 ms; *n* = 7, *P* > 0.05). However, at higher frequencies (100 Hz) DHK slowed down the decay of the transients by 45% compared with 50 Hz stimulation (100 Hz baseline: *τ*_decay_ = 86.23 ± 7.5 ms, 100 Hz DHK: *τ*_decay_ = 123.85 ± 8.66 ms; *n* = 7, ***P < 0.0001). Consistently, astrocyte patch-clamp recordings showed that in the presence of DHK, STCs evoked at 100 Hz become slower than those evoked at 50 Hz (Supplementary Fig. 7). This suggests that GLT-1 transporters are required to sustain highly efficient glutamate uptake at particularly high-frequency stimulation (100 Hz).

**Fig. 7:**
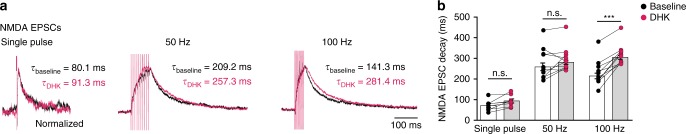
Effect of DHK on NMDA current kinetics in the ACC. **a** Representative traces of NMDA currents (layer 1 synaptic stimulation) normalized to the peak amplitude recorded from layer 5 pyramidal cells in the ACC following single pulse, trains of ten pulses at 50 and 100 Hz (extracellular synaptic stimulations) at baseline (black traces) and in the presence of the GLT-1 antagonist DHK (250 μM, purple traces). Holding potential at + 40 mV. **b** The decay kinetics of NMDA-evoked EPSCs are slightly but not significantly slower in the presence of DHK at single pulse (baseline: 72.3 ± 9.2 ms; DHK: 93.9 ± 9.9 ms, *n* = 8, *P* > 0.05) and 50 Hz stimulations (baseline: 259.1 ± 18.6 ms; DHK: 281.4 ± 10.1 ms, *n* = 9, *P* > 0.05). However, at 100 Hz, the decay kinetics become significantly slower in the presence of DHK (baseline: 215 ± 13.6 ms; DHK: 305 ± 9.1 ms, *n* = 9, *N* = 3, *** *P* < 0.001). *n* = number of cells, *N* = number of mice. Data are mean ± SEM. Two-way RM ANOVA test

We then tested the effect of DHK on NMDA currents recorded from L5 pyramidal cells of the ACC (layer 1 stimulation, Fig. 7). Consistent with its effect on the time course of synaptically released glutamate (Fig. 6), DHK significantly slowed the NMDA current kinetics at the 100 Hz synaptic stimulation paradigm (Fig. 7b, 100 Hz: baseline 215 ± 13.6 ms; DHK 305 ± 9.1 ms, *n* = 9, *** *P* < 0.001), while it exerted only a minor effect at lower stimulation frequencies (Fig. 7b, single pulse: baseline 72.3 ± 9.2 ms; DHK 93.9 ± 9.9 ms, *n* = 8, *P* > 0.05 and at 50 Hz: baseline 259.1 ± 18.6 ms; DHK 281.4 ± 10.1 ms, *n* = 9, *P* > 0.05).

### The role of non-GLT-1 transporters in glutamate clearance

Interestingly, in situ hybridization studies revealed strong GLAST expression in the upper layers of the frontal cortex^37^ but the functional significance of such high expression has never been investigated. To directly test the relative enrichment of GLAST and GLT-1 in this brain area, we performed western blot analysis of the two GluTs in the frontal cortex and in the barrel cortex of the same animals (Supplementary Fig. 8c–e). Our results revealed a strong expression of both GLT-1 and GLAST in the two cortical regions, GLAST being more enriched in the ACC relative to the BC. Consistently, quantitative RT-PCR analysis showed that GLAST/GLT-1 ratio is significantly higher in the frontal cortex compared with the barrel cortex (Supplementary Fig. 8b).

**Fig. 8:**
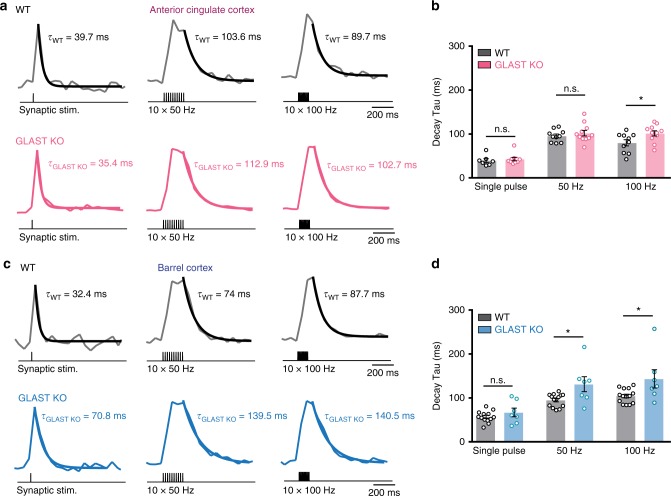
Temporal profile of extrasynaptic glutamate clearance is slower in GLAST KO mice in the ACC and BC. **a** Example traces of iGluSnFr responses in the anterior cingulate cortex (ACC) following synaptic stimulation (single stimulation, 10 × 50 Hz and 10 × 100 Hz) in WT mice (black traces) and GLAST KO mice (GLAST^CreERT2/CreERT2^ pink traces). **b** In the ACC, GLAST KO mice show slower kinetics of the events only at 100 Hz compared with WT mice (*n*_WT_ = 10, *n*_GLAST KO_ = 11, *N*_WT_ = 6, *N*_GLAST KO_ = 3; *P*_single_ = 0.58, *P*_50 Hz_ = 0.42, *P_100 Hz_ = 0.02). **c** Same as (**a**) but in the BC. **d** In the BC, GLAST KO mice show slower kinetics of the events at both 50 Hz and 100 Hz compared with WT mice (*n*_WT_ = 14, *n*_GLAST KO_ = 7, *N*_WT_ = 6, *N*_GLAST KO_ = 3; *P*_single_ = 0.26, *P_50 Hz_ = 0.01, *P_100 Hz_ = 0.02). *n* = number of slices, *N* = number of mice. Data are mean ± SEM. Two-tailed unpaired *t* test

Since GLAST has been reported to possess higher affinity for glutamate compared with GLT-1^38^, although this depends on the detection method^39^, we reasoned that such a transporter, if sufficiently expressed, could account for a substantial amount of glutamate uptake. To directly assess the role of GLAST in glutamate uptake in the ACC, we decided to take advantage of GLAST functional knock-out (KO) mice^40^. We found no differences in the decay kinetics of glutamate transients in GLAST KO mice compared with WT mice at single pulse and 50 Hz stimulation (Fig. 8a, b). These results suggest that, despite the high GLAST expression in WT mice (Supplementary Fig. 8), in GLAST KO mice, non-GLAST GluTs (likely GLT-1) are sufficient to compensate for the absence of GLAST in the ACC for synaptic stimulations up to 50 Hz. However, at 100 Hz, glutamate transients in the ACC of GLAST KO mice were significantly slower than in the WT mice (Fig. 8a, b; single pulse: WT 39.5 ± 4.5 ms, GLAST KO 42.7 ± 3.7 ms; 50 Hz: WT 95.5 ± 4.3 ms, GLAST KO 101.9 ± 6.3 ms; 100 Hz: WT 79.9 ± 7.1 ms, GLAST KO 101.5 ± 5.9 ms; *n*_WT_ = 10; *n*_GLAST KO_ = 11, *P*_single_ = 0.58, *P*_50 Hz_ = 0.42, *P_100 Hz_ = 0.02). These results demonstrate that GLAST together with GLT-1 (Fig. 6) are important to maintain efficient glutamate uptake capacity at high-frequency synaptic stimulation in the ACC.

GLT-1 is massively expressed in the adult somatosensory cortex^35,36^. However, recent findings suggest that GLAST expression levels are also very high^20^ and that both GLT-1 and GLAST appear among the 20 most expressed astrocytic genes in the sensory cortex^41^. Our western blot and qRT-PCR experiments also indicate a high expression of GLAST in the BC (Supplementary Fig. 8). This pronounced expression suggests that GLAST may also play a role in glutamate uptake in the somatosensory cortex. Hence, we tested glutamate clearance in BC of functional GLAST KO mice. We found that glutamate transients are significantly slower in the BC of these mice compared with WT animals at high-frequency stimulation (50 and 100 Hz) but not at single pulse (Fig. 8c, d; single pulse: WT 56.9 ± 3.6 ms, GLAST KO 66.9 ± 9.8 ms; 50 Hz: WT 94.8 ± 3.9 ms, GLAST KO 131.2 ± 16.9 ms; 100 Hz: WT 104.3 ± 4.3 ms, GLAST KO 143.3 ± 20.7 ms; *n*_WT_ = 14; *n*_GLAST KO_ = 7, *P*_single_ = 0.26, *P_50 Hz_ = 0.01, *P_100 Hz_ = 0.02). Hence, these data demonstrate that GLAST is also involved in glutamate uptake in the BC.

**Fig. 9:**
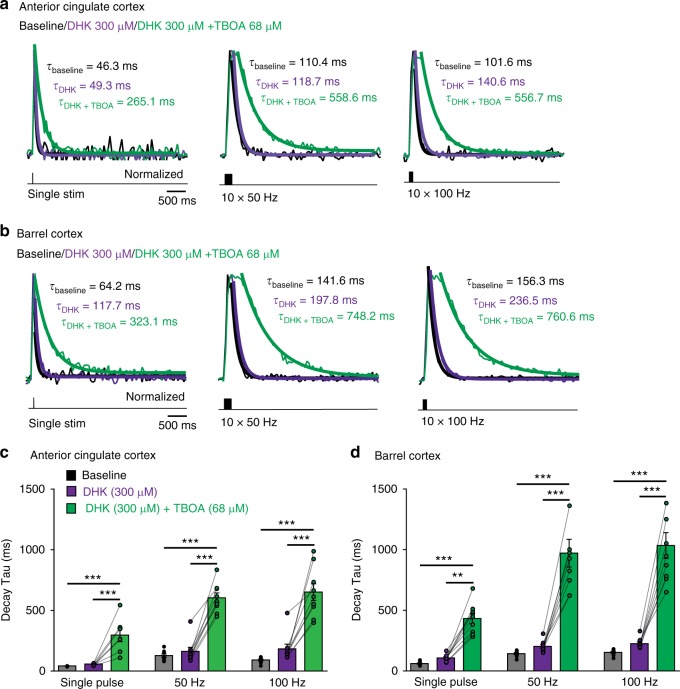
Contribution of GLT-1 and non-GLT1 transporters to glutamate uptake. **a** Example traces of iGluSnFr responses in the ACC following synaptic stimulation (single stimulation, 10 × 50 Hz and 10 × 100 Hz) during baseline (black traces), in the presence of 300 µM DHK, which completely blocks GLT-1 (purple traces) and in the presence of both DHK 300 µM and 68 µM DL-TBOA (green traces), which also blocks about 50% of GLAST. Thick lines are mono-exponential fits. **b** Similar to (**a**) but in the BC. **c** In the ACC, blocking about 50% of GLAST in addition to GLT-1 (green bars) strongly slows the kinetics of the events at all stimulations compared with only blocking GLT-1 by DHK (purple bars; slowdown by 413% for single stim, by 271% for 50 Hz and by 255% for 100 Hz; *n* = 9, *N* = 3, ***P_single_ < 0.001, ***P_50 Hz, 100 Hz_ < 0.0001). **d** In the BC, blocking 50% of GLAST in addition to GLT-1 (green bars) strongly slows the kinetics of the events at all stimulation frequencies compared to only blocking GLT-1 by DHK (purple bars; slowdown by 300% for single stim, by 380% for 50 Hz and by 360% for 100 Hz; *n* = 9, *N* = 3, **P_single_ < 0.01, ***P_50 Hz, 100 Hz_ < 0.0001). Please note the different time scale compared to the other figures. Traces normalized to the peak. *n* = number of slices, *N* = number of mice. Data are mean ± SEM. Two-way RM ANOVA test

Since both GLT-1 and GLAST appear to contribute to glutamate uptake, we further tested whether blocking both GLT-1 and non-GLT-1 transporters simultaneously would have a stronger impact on glutamate clearance. Therefore, we applied 68 µM DL-TBOA (broad spectrum GluT blocker) in the presence of the GLT-1 antagonist DHK. This drug combination is expected to block GLT-1, a fraction of GLAST and possibly other non-GLT-1 transporters^42^. As expected, the decay of synaptically evoked iGluSnFr signals in the ACC were dramatically increased (Fig. 9a, c; single pulse: DHK 57.86 ± 3.3 ms, DHK + TBOA 297 ± 54 ms, 413% increase; 50 Hz: DHK 183.4 ± 38.4 ms, DHK + TBOA 604.5 ± 40 ms, 271% increase; 100 Hz: DHK 163 ± 32.6 ms, DHK + TBOA 651.5 ± 68.3 ms, 255% increase; *n* = 9, ***P_single_ < 0.001, ***P_50 Hz, 100 Hz_ < 0.0001).

We then performed similar experiments in the BC by first blocking GLT-1 with DHK and subsequently blocking non-GLT-1 transporters with 68 µM DL-TBOA. DL-TBOA slowed down synaptically evoked glutamate signals in the BC by ~350% (Fig. 9b, d, single pulse: DHK 107.68 ± 9.5 ms, DHK + TBOA 432.7 ± 42 ms, 302% increase; 50 Hz: DHK 203.3 ± 15.3 ms, DHK + TBOA 971.3 ± 114.1 ms, 378% increase; 100 Hz: DHK 225.9 ± 14.6 ms, DHK + TBOA 1034 ± 106.3 ms, 358% increase; *n* = 9, **P_single_ < 0.01, ***P_50 Hz, 100 Hz_ < 0.0001). Thus, in agreement with recent reports^43^, blocking both GLT-1 and a fraction of non-GLT-1 transporters (including GLAST) strongly reduced glutamate uptake also in the BC. Interestingly, full blockade of all GluTs with 300 µm DL-TBOA leads to similar synaptically evoked iGluSnFR signals in the ACC and BC, suggesting that glutamate time course in the absence of active uptake is the same in both regions (Supplementary Fig. 9).

Altogether, our data suggest that in the ACC, during high-frequency synaptic stimulation (100 Hz), both GLT-1 and non-GLT-1 transporters are required to shorten the lifetime of extrasynaptic glutamate, thereby preventing neuronal over-excitation. On the other hand, in the barrel cortex, glutamate uptake slows down at increasing stimulation frequencies (100 Hz compared with 50 Hz) and both GLT-1 and non-GLT-1 transporters (including GLAST) contribute to glutamate uptake upon both temporally sparse and sustained synaptic stimulations. These findings point to a striking difference in glutamate uptake between two cortical regions and are a good example of circuit-specific astrocyte specialization and of how this may impact neuronal function.

## Discussion

Glial GluTs play a vital role in the regulation of glutamate time course at synaptic and extrasynaptic sites. It has long been believed that the astrocyte glutamate uptake system, being far from saturation, does not vary when neuronal activity is sustained^44^. Recently, studies performed in the somatosensory cortex revealed that during high-frequency synaptic activity, the astrocyte glutamate uptake system reduces its capacity, leading to enhanced activation of NMDA receptors^10^ and promoting pathological cortical spreading depression^12^. We found that the frontal cortex is equipped with a glutamate uptake system that not only can handle high-frequency synaptic discharges, but can also rapidly increase its clearance capacity when challenged with a higher-frequency synaptic glutamate release. In addition, we report that, contrary to the somatosensory cortex and the hippocampus, where GLT-1 contributes to astrocyte-mediated glutamate uptake^35,36^, specific blockade of GLT-1 in the frontal cortex does not influence the lifetime of extrasynaptic glutamate for synaptic stimulations up to 50 Hz. GLAST and GLT-1 (and possibly other transporters) instead act in concert during 100 Hz synaptic discharges to improve glutamate uptake and limit neuronal NMDAR activation. The combined glutamate uptake by GLT-1 and non-GLT-1 transporters is expected to lead to particularly high intracellular Na^+^ increase in ACC astrocytes. Such Na^+^ increase would likely trigger a strong activation of the energy-consuming NKA, a pathway that boosts astrocyte aerobic glycolysis and lactate production necessary for memory formation^32,45^. Accordingly, in the adult human brain, aerobic glycolysis accounts for up to 25% of glucose utilization in the ACC while being less prominent in other brain regions^46^. Consistent with this notion, we found that blocking NKA activity with ouabain 5 µM strongly slowed down the decay kinetics of iGluSnFr-mediated glutamate signals at all synaptic stimulation intensities, confirming the tight coupling between NKA activity and the astrocyte glutamate clearance system^47^. This effect is likely to be ascribed to the inhibition of α2 NKA, which is highly expressed by mature astrocytes^29,31^.

An additional difference between ACC and BC was revealed when comparing iGluSnFr decay Tau upon 50 Hz synaptic stimuli, which appeared to be slower in the ACC compared with the BC (Fig. 2). A similar trend was detected upon trains of short puffs of exogenous glutamate at 50 Hz (Fig. 3). One possibility is that the combined contribution of GLT-1 and non-GLT-1 transporters to glutamate uptake at 50 Hz in the BC would improve glutamate clearance. Since iGluSnFr decay kinetics are faster in the ACC compared with the BC for 100 Hz synaptic stimulations, it is intriguing to speculate that the glutamate uptake machinery of the two brain regions is preferentially tuned to specific stimulation frequencies.

Other than with glutamate transporters, astrocytes may differentially control the time course of synaptically released glutamate uptake by modulating glutamate diffusion^48^. Hence, perisynaptic astrocyte processes represent a physical barrier that hinders the movement of transmitters in the tissue^48^. Regional differences in astrocyte to post-synaptic density distances (affecting neuropil tortuosity and glutamate diffusion coefficient^49^) may therefore participate in the differential NMDAR activation in the two cortical regions.

The reasons for the different glutamate uptake properties between BC and ACC are at present unclear. One possibility is that astrocytes are patterned during development to display circuit-specific features^50^. Alternatively, astrocytes could modify their function according to the level of activity of the neuronal network^11,51^ for instance by increasing their GluT expression^45,52^. Hence, astrocytes, when confronted with persistent neuronal activity (a distinguishing feature of frontal cortex networks), may enhance their glutamate uptake in order to prevent circuit hyperexcitability, seizures, and CSD. Conversely, since the primary somatosensory network is characterized by sparse action potential firing^53^, astrocytes in this cortical area may adapt their function according to this low network activity regime.

Is neuronal network function affected by such inter-regional differences? We show that, in the frontal cortex, glutamate uptake limits NMDAR activation during high-frequency (100 Hz) synaptic activity. Interestingly, 100 Hz synaptic stimulation paradigms have been widely used to induce long-term synaptic potentiation (LTP). Since NMDAR function is crucial for LTP, the enhanced glutamate uptake in the frontal versus somatosensory cortex could influence the threshold for the induction of such plasticity. Accordingly, enhanced GluT function and astroglial synaptic coverage have been shown to impair LTP^54–56^ and disrupt the rules for spike-timing-dependent synaptic plasticity^57^. In addition, NMDAR activation at the apical dendrites of pyramidal cells in the somatosensory cortex has been shown to be pivotal for the initiation of NMDA-dependent dendritic spikes, i.e., large step-like increases in synaptic responses at dendrites that promote neuronal firing^58,59^. Our dendritic recordings predict that the likelihood of initiation of NMDA-dependent dendritic spikes will be higher in S1 compared with the frontal cortex, since in the latter brain region highly effective glutamate uptake limits NMDAR activation. In agreement with this hypothesis, previous work has shown that partial blockade of glutamate uptake strongly promotes the generation of NMDA-dependent dendritic non-linearities in the frontal cortex^5^, suggesting that GluTs may limit dendritic spike generation in this brain region.

It has been shown that generalized dysfunction of the glutamate uptake system generates circuit-specific alterations in pathological conditions such as Huntington’s disease^60^, astrogliosis-induced epilepsy^61^, and familial hemiplegic migraine type 2^12,62^. It is intriguing to speculate that regional differences in glutamate uptake may render specific circuits more susceptible to pathological alterations. For instance, the threshold for cortical spreading depression (CSD) initiation is different in individual cortical regions^63^ and brain region-specific GLT-1 deletion differentially affect the occurrence of epileptic seizures^64^. In addition, as distinct categories of astrocytes respond differently to brain injury and inflammation and exert opposite effects on neuronal survival^65,66^, it is possible that such differences may at least partially rely on the efficacy of the astrocytic glutamate transport system. In conclusion, our data support and further extend the growing notion that astrocytes adapt to specific circuits, which in turn shape their function. This functional specialization may therefore deeply impact the excitability of neuronal networks and could help to clarify the nature of astrocyte-mediated pathological circuit-specific dysfunctions.

## Methods

### Animals

Permission for animal experiments was obtained from the Tierversuchskommission of the canton of Zurich, Zurich, Switzerland. Adult mice were group housed in filtered cages with a standard 12-h light/dark cycle and food and water available ad libitum. Mice were housed with a maximum of five animals per cage.

Homozygous GLAST-CreERT2 knock-in mice^40^ have been used as functional GLAST-KO mice. The GLAST-CreERT2 knock-in produces a GLAST-KO allele, as previously shown by western blot and mRNA analysis^67^. These functional GLAST KO mice were generated by crossing heterozygous GLAST ^CreERT2/+^ mice and litters homozygous for GLAST ^CreERT2/CreERT2^ were used for the study. Genotypes were determined by PCR using the following three primers: GAG GCA CTT GGC TAG GCT CTG AGG A, GAG GAG ATC CTG ACC GAT CAG TTG G, and GGT GTA CGG TCA GTA AAT TGG ACA T. The GLAST wild-type and knock-in alleles produce a PCR product of 700 and 400 bp, respectively.

### Acute brain slice preparation

Five to eight-week-old C57BL/6J male and female mice were briefly anesthetized with isoflurane and decapitated. The brain was quickly removed and transferred to ice-cold solution containing (in mM) 65 NaCl, 2.5 KCl, 1.25 NaH_2_PO_4_, 25 NaHCO_3_, 7 MgCl_2_, 0.5 CaCl_2_, 25 glucose, and 105 sucrose saturated with 95% O_2_ and 5% CO_2_; 350-μm-thick coronal slices containing the ACC or the barrel cortex were cut from the tissue block with a vibratome (HM 650, Microm). Slices were then transferred to a recovery solution containing (in mM) 130 K-gluconate, 15 KCl, 0.2 EGTA, 20 HEPES, 25 glucose for 2 min before being kept in oxygenated artificial cerebrospinal fluid (ACSF, 315 mOsm) saturated with 95% O_2_ and 5% CO_2_ and containing (in mM) 125 NaCl, 2.5 KCl, 1.25 NaH_2_PO_4_, 25 NaHCO_3_, 1 MgCl_2_, 2 CaCl_2_, and 25 glucose at 34 °C for 25 min and then at room temperature until use. Slices used for two-photon glutamate imaging and astrocytic patch-clamp recordings were loaded with sulforhodamine 101 dye (SR-101, 1 μM) for 15 min at 34 °C before being kept in ACSF at room temperature. The impact of SR-101 incubation on our experiments is reported in Supplementary Fig. 2.

### Chemicals and drugs

Reagents for ACSF and internal solutions, biocytin, NBQX, BaCl_2_, and picrotoxin were obtained from Sigma-Aldrich. CNQX, AP-5, DHK, DL-TBOA, TTX, ouabain, and D-serine were obtained from Tocris. L-glutamic acid from BioTrend and SR-101 from Invitrogen. NBQX, CNQX, and DL-TBOA were dissolved in DMSO. Picrotoxin was dissolved in EtOH. AP-5, D-serine, TTX, ouabain, and DHK were dissolved in ddH_2_O.

In both patch-clamp and two-photon imaging experiments, following baseline recordings, drugs were applied in the external solution for at least 15 min prior to recordings. For the double pharmacology imaging experiments (Fig. 9) following baseline recordings, DHK (300 μM) was first applied and recordings were acquired 20 min later. Subsequently, DHK (300 μM) and DL-TBOA (68 μM) were applied for 20 min before recording. In a number of recordings in which we applied much higher doses of DL-TBOA (300 µM), we observed a change in baseline iGluSnFr fluorescence (similar to ref. ^18^), a reduced amplitude of the evoked responses and cellular swelling often accompanied by a lateral or Z drift. These experiments had to be excluded, which explains the low *n* value for this set of experiments (Supplementary Fig. 9).

### Cloning and virus production

The EGFP gene was cloned into a plasmid backbone containing a shortened glial fibrillary acidic protein (HgfaABC1D) promoter as in ref. ^68^. These plasmids were packaged into AAV serotype 5 (ssAAV5/2-hGFAP-hHBb1/E-EGFP-bGHp(A)) by the Viral Vector Facility of the University of Zurich. Plasmids information is available at viral vector repository of the viral vector facility of the University of Zurich https://vvf.ethz.ch.

The virus containing the iGluSnFr (AAV2/1.GFAP.iGluSnFr.WPRE.SV40) is from Penn Vector and was provided by Dr. Loren Looger, Janelia Farm.

### Astrocytic whole-cell patch-clamp recordings

Individual slices were transferred to a recording chamber perfused with oxygenated ACSF, at a flow rate of 1–2 mL min^-1^. All electrophysiology experiments were performed at 32–34 °C. Whole-cell recordings were taken from layer 1 astrocytes in the ACC and in the barrel cortex. Unless otherwise stated, cell bodies of astrocytes were visualized using an astrocyte-specific dye (SR-101, 1 μM) that was visualized with wLS broad-band LED illumination (460 nm) and images were acquired with Retiga R1 camera using Ocular software (Qimaging, Germany) through a ×40 water immersion objective. In addition, astrocytes were recognized by their hyperpolarized resting membrane potential, their linear current–voltage relationships, their inability to generate action potentials and their low-input resistance. Astrocytes were patched with borosilicate glass pipettes (4–8 MΩ) containing the following internal solution (in mM) 115 K-gluconate, 6 KCl, 5 glucose, 7.8 Na-phosphocreatine, 4 Mg-ATP, 0.4 Na-GTP, pH 7.25 with KOH, osmolarity 295 mOsm (readjusted with sucrose when necessary). Recordings were performed using Multiclamp 700B amplifier and data were acquired with a Digidata 1550A 16-bit board (all from Molecular Devices). For the recordings of synaptically mediated glutamate transporter currents, the extracellular solution contained antagonists of NMDA receptors (AP-V, 50 μM), AMPA receptors (CNQX or NBQX, 10 μM), and GABA_A_ receptors (Picrotoxin 100 μM). Astrocytes were held at −80 mV, and synaptically activated glutamate transporter currents (STCs) were evoked by single-pulse stimulation or by trains of 11 pulses at high frequencies (50 Hz then 100 Hz) every 20 s. Every protocol was repeated at least five times and then averaged and analyzed. Currents were evoked by focal electrical synaptic stimulation (bipolar, 100 μs, 8.5 V) through a theta-glass pipette placed in layer 1, in proximity to the recorded astrocyte. Access resistance was monitored (<16 MΩ) and recordings with an access resistance changing more than 30% between the beginning and the end of the recording were discarded. Resting membrane potential and input resistance were monitored for analysis of electrophysiological properties of astrocytes. The decay kinetics of the last pulse of the trains were analyzed by subtracting the current elicited by 10 pulses from the current elicited by the 11th pulse. We attempted to pharmacologically isolate STCs with DL-TBOA. We realized that in our current preparation (i.e., adult cortical slices), saturating concentrations of DL-TBOA often resulted in unstable recordings leading to a major membrane depolarization sometimes accompanied by cellular swelling. Thus, subtraction of the small (5–10 pA) sustained K^+^ component was not possible for single-pulse stimulations. To visualize astrocytes for targeted patch-clamp recordings without incubation in SR-101, 2 weeks prior to the experiment we injected in the ACC 0.5 μl of an adeno-associated virus driving the expression of the fluorescent protein EGFP in astrocytes, i.e., under the astrocyte-specific promoter GFAP.

### Recordings of synaptic currents

Whole-cell patch-clamp recordings were taken from the soma and apical dendrites of layer 5 pyramidal cells in the barrel cortex or the ACC. Recordings were made with the same electrophysiological setup used for astrocytic whole-cell patch-clamp experiments. Individual slices were put in a recording chamber with ACSF. Somata were patched with borosilicate glass pipettes (2.5–4 MΩ). For AMPA-mediated EPSCs, cells were voltage clamped at −70 mV and stable AMPA-mediated synaptic responses were recorded in the presence of AP-5 (50 μM) and picrotoxin (100 μM). As for NMDA-mediated EPSCs, cells were first voltage clamped at −70 mV, and subsequently clamped at + 40 mV to relieve NMDA receptors from the Mg^2+^ block and CNQX or NBQX (10 μM) were applied to block AMPA receptors in the presence of picrotoxin (100 μM) and D-serine (10 μM). AMPA and NMDA receptor-mediated currents were evoked in the same way as STCs. Series resistance was compensated if necessary (up to 15%). Recordings with an access resistance >15 MΩ were discarded. The following internal solution was used for somatic recordings (in mM): 130 cesium gluconate, 5 CsCl, 10 Na-HEPES, 1.1 EGTA, 10 phosphocreatine, 4 Mg-ATP, 0.3 GTP, and biocytin (1.5 mg mL^−1^), pH 7.3 with CsOH, osmolarity 297 mOsm. L5 pyramidal cells with thick apical dendrites were selected in the barrel cortex for dendritic whole-cell patch-clamp recordings. Apical dendrites of L5 pyramidal cells in the barrel cortex were patched (80–200 μm from soma) with small thick-wall borosilicate pipettes (16–21 MΩ) containing the following internal solution (in mM): 115 K-gluconate, 20 KCl, 2 Mg-ATP, 2 Na-ATP, 10 Na-phosphocreatine, 0.3 Na-GTP, 10 HEPES, and biocytin (1.5 mg mL^−1^), pH 7.3 with KOH, osmolarity 292 mOsm. Electrode capacitance was compensated and once a gigaseal was obtained, whole-cell configuration was achieved by hyperpolarization. Subsequently, dendrites were clamped at least at + 60 mV to relieve NMDA receptors from the Mg^2+^ block, i.e., until a clear positive current was evoked. Series resistance was compensated (up to 50%) at the beginning of the recording and during the recording if required. Recordings were taken in the presence of CNQX (10 μM), D-serine (10 μM), and picrotoxin (100 μM). NMDA receptor-mediated currents were evoked in the same way as STCs. Please note that differences between the dendritic and somatic NMDAR decay might be due to the different intracellular solutions, which likely influences the membrane time constant.

### Glutamate imaging

Five to six-week-old mice were injected with 0.3–0.5 μl of AAV2/1.GFAP.iGluSnFr.WPRE.SV40 (Penn Vector; provided by Dr. Loren Looger, Janelia Farm) unilaterally into the BC or ACC through a glass pipette. Stereotaxic co-ordinates with respect to bregma were (in mm) 1 posterior, 3.5 lateral, 0.3 ventral for the BC and 0.75 anterior, 0.3 lateral, and 1.25 ventral for the ACC. The same coordinates were used for experiments presented in Supplementary Fig. 2. Fifteen to eighteen days following the viral injections, coronal brain slices (350 μm) containing the BC or the ACC were obtained as described above^13^. A galvanometer-based two-photon laser scanning system was used to image extrasynaptic glutamate (×16 objective, zoom 6, excitation wavelength 900 nm, 64 × 64 pixels/image, acquisition rate 19.2 Hz). Astrocytes were visualized using SR-101.

Synaptic glutamate release: Synaptic glutamate release was elicited by single pulse stimulation and by trains of ten pulses at high frequency (10, 50, or 100 Hz) every 20 s delivered via a theta-glass pipette (bipolar, 100 μs, stimulation intensity 3–5 V) placed in the inner layer 1. In order to visualize the theta-glass pipette, the latter was filled with ACSF containing 1 μM SR-101. All solutions contained NBQX or CNQX 10 μM, APV 50 μM, picrotoxin 100 μM, and temperature was maintained between 32 and 34 °C while imaging. Ten consecutive sweeps were acquired and subsequently analyzed using ImageJ. Fluorescence emission was collected from a region of interest (diameter 34 μm) 10–40 μm away from the stimulation pipette. The ROI size was selected according to the following criteria: (a) a region distal enough for the simulation point but not too far that no glutamate was detected (typical radius of our signal upon a single stimulation was 70–80 µm from the pipette; (b) a ROI size large enough to allow averaging signals from as many pixels as possible (177 pixels) but without taking into consideration large sub-regions that show no signal (which would reduce the signal amplitude). The average background value was determined from a region within the field of view that was free of clearly visible iGluSnFr (typically outside the slice for the BC and in the contralateral hemisphere for the ACC) and subtracted from the fluorescence intensity of the ROIs for each frame. Traces were then averaged and decay Tau was calculated by fitting a single exponential function using Igor Pro (wavemetrics). In the few cases in which the transients appeared to display a (possible) second component, a two-term fit was applied. For this we have been mindful of the R^2^ (r-square) values of the single- and two-term fits and we applied an F-test to determine whether a second-term was needed to explain a significant portion of the data. In a few cases, the traces could also be described with a double exponential. Yet, in these cases the time constant of the first term was almost identical to the one obtained by fitting the trace with a single exponential. Conversely, the second component was typically at least one order of magnitude larger (in the seconds range), hence incompatible with a signal detecting residual glutamate in the extracellular space. We therefore decided to maintain the mono-exponential fit, also for consistency with the rest of the data and the published literature^10,16,18^. In agreement with previous reports^18^, we verified that the decay Tau was independent of the ROI location and relative distance from the stimulation pipette (Supplementary Fig. 3), suggesting that the iGluSnFR response is unlikely to represent glutamate diffusion originating from an area of maximal release.

Exogenous glutamate puff: L-glutamic acid (1 mM) diluted in ACSF was locally applied via a thin-walled borosilicate pipette (0.69 × 1.20 × 80 mm, with filament, Science Products *GmbH*) placed in layer 1 of the BC or of the ACC. Using an externally triggered pressure ejector (Toohey Company, Fairfield, New Jersey) connected to the puff pipette, we applied trains of ten short glutamate puffs of 8 ms at 50 and 100 Hz with a pressure of 24 ± 4 PSI or continuous puffs of 100 and 200 ms with an average pressure of 14 ± 4 PSI. For the 50 and 100 Hz puffs, we first applied a pressure of 20 PSI, if a response was not observed we gradually increased the pressure until we could see a response. The same was done for the continuous puffs, starting with a pressure of 10 PSI. All solutions contained CNQX 10 μM, TTX 1 μM, picrotoxin 100 μM and temperature was maintained between 32 and 34 °C while imaging. Ten consecutive sweeps were acquired and subsequently analyzed using ImageJ. Fluorescence emission was collected from a region of interest (diameter 15.9 μm) 10–40 μm away from the stimulation pipette. Images and decay kinetics were then analyzed as described above for the synaptic glutamate release imaging experiments. The pipette was also filled with 1 μM SR-101 for visualization.

Higher resolution images (526 × 526 pixels/image) of layer 1 were obtained for the two-photon system to calculate the cell body area and number of astrocytes using ImageJ.

### Biocytin labeling

For some experiments, internal solutions used for astrocytic and somatic L5 pyramidal cells patch-clamp experiments contained biocytin (1.5 mg ml^−1^) that diffused in the cells for at least 10 min. Slices (350 μm) containing the recorded cell were then immersion fixed in 4% PFA at 4 °C overnight. The following day, slices were washed in PBS 274 mM NaCl before being transferred to blocking solution containing 10% NGS in 0.3% Triton-PBS 274 mM NaCl for 1 h. Afterward, for the immunochemical staining, slices were put in blocking solution containing Alexa Fluor 647-conjugated streptavidin (1:700, Jackson ImmunoResearch Europe Ltd, code: 016-600-084) for 2 h. Slices were then washed three times for 10 min each in PBS containing 274 mM NaCl before being mounted on superfrost plus microscope slides. Images were acquired on a Zeiss LSM710 Pascal confocal microscope through a 0.9 NA × 10 Plan-apochromat objective (for L5 pyramidal cells) or a 1 NA × 20 Plan-apochromat objective (for astrocytes labeling) and the ZEN2012 software (Carl Zeiss). Whenever applicable, contrast and illumination were adjusted in ImageJ. Presented images are Z projections.

### Western blot analysis

For western blot analysis, the upper layers of ACC and BC were carefully isolated from 1-mm-thick coronal brain slices obtained from adult C57BL/6J mice. The tissue was immediately snap-frozen on dry ice and stored at −80 °C until used. On the day of the experiment, the tissue was thawed and homogenized in 150 μl buffer (20 mM Tris pH 7.4, 5 mM NaF, 1 mM EDTA, 1 mM EGTA plus the protease inhibitor cocktail CompleteMini (Sigma-Aldrich)) by sonication. After determining protein concentration using the Bradford protein assay (BioRad), the samples were incubated with Laemmli sample buffer (BioRad) for 2 h at 37 °C, and aliquots containing 20 μg of protein were separated by sodium dodecyl sulfate-polyacrylamide gel electrophoresis (SDS-PAGE) using 10% mini-gels (Mini Protean 3, BioRad). Proteins were transferred onto nitrocellulose membranes in a Mini Trans-Blot Module (BioRad) at 365 mA for 90 min using 192 mM glycine, 25 mM Tris, 0.1% SDS, 20% methanol as transfer buffer. After blotting, the transferred proteins were stained with Amidoblack and immediately imaged using the E-box VX2 gel imager (Vilber). For immunodetection, the blots were blocked for 1 h in PBST (PBS pH 7.4, 0.05% Tween 20) containing 5% n-fat dry milk at room temperature, followed by incubation overnight at 4 °C with anti-GLT1 antibodies (1:700, rabbit polyclonal, knock-out verified, Synaptic Systems, Cat. No. 250203) or anti-GLAST antibodies (1:1500, rabbit polyclonal, knock-out verified, Synaptic Systems, Cat. No. 250113), diluted in PBST containing 5% non-fat dry milk. The blots were then washed five times for 10 min with PBST and incubated with secondary antibodies conjugated to horseradish peroxidase for 1 h at room temperature. Following extensive washing (see above), immunoreactivity was detected by the chemoluminescence method (SuperSignal West Dura, Thermo Scientific) using a Fujifilm LAS-1000 imager. Immunoreactivity was quantified with the AIDA software (Raytest) and normalized to total protein in the corresponding lanes (determined by Amidoblack staining, see above).

### qRT-PCR

Fresh tissues from adult C57BL/6J mice (*N* = 6 animals) of the ACC and the BC were dissected from the tissue block with a vibratome (HM 650, Microm) in an ice-cold solution. The total purified RNA was isolated from the fresh tissues using the RNeasy Plus Micro Kit (Qiagen, Hilden, Germany) in accordance with the manufacturer’s protocol. The concentration and purity of the RNA samples were determined using a spectrophotometer. Total RNA (1 μg) was reversely transcribed (RT) to c-DNA using the QuantiTect Reverse Transcription Kit (Qiagen, Hilden, Germany) following the manifacturer’s protocol. Real-time quantitative PCR (qPCR) was performed following reverse transcription using 5X HOT FIREPol EvaGreen qPCR Mix Plus (Solis Biodyne, Tartu, Estonia). qPCR was carried out in technical triplicates on a 384-well plate in the 7900HT Fast real-time PCR System (Applied Biosystems, Foster City, USA). β2 microglobulin (B2M), beta**-**actin (ACT B), and cytochrome C1 (CYC1) were used as internal controls (Microsynth, Balgach, Switzerland). The primer sequences are listed in Supplementary Table 1. We performed standard curves in order to determine the efficiency for all of the genes. The relative gene expression levels of *Slc1a2* (GLT-1) and *Slc1a3* (GLAST) compared with the internal controls were calculated using the 2^−ΔCT^ method^69^ and the relative gene expression levels in the ACC compared with the BC were calculated using the 2^−ΔΔCT^ method^69^. The ratio of GLAST/GLT-1 in the ACC compared with the BC were calculated using the 2^−ΔCT^ method.

### Statistics

All data are displayed as the mean ± SEM. Statistical comparisons were made with two-tailed paired, unpaired *t* tests, one-way ANOVA with Bonferroni post hoc test, or two-way RM ANOVA test. All graphs and statistical tests were made in Graphpad prism and figures were prepared using Adobe Illustrator CS5. *P* values <0.05 were considered statistically significant.

### Reporting summary

Further information on experimental design is available in the Nature Research Reporting Summary linked to this article.

## Supplementary information

Supplementary Information

Reporting Summary

## Data Availability

Data, associated protocols and additional information regarding this paper are available to the reader upon request from the authors and are located on the internal server of the institute of Pharmacology and Toxicology at the University of Zurich.
