# Supplementary material for: Differences in glutamate uptake between cortical regions impact neuronal NMDA receptor activation
Source: Commun Biol. 2019 Apr 5;2:127. doi: 10.1038/s42003-019-0367-9 (PMC6451009; doi:10.1038/s42003-019-0367-9)
Supplement: Supplementary file 1 — Supplementary Information [file 42003_2019_367_MOESM1_ESM.docx]

Supplementary Figures

**
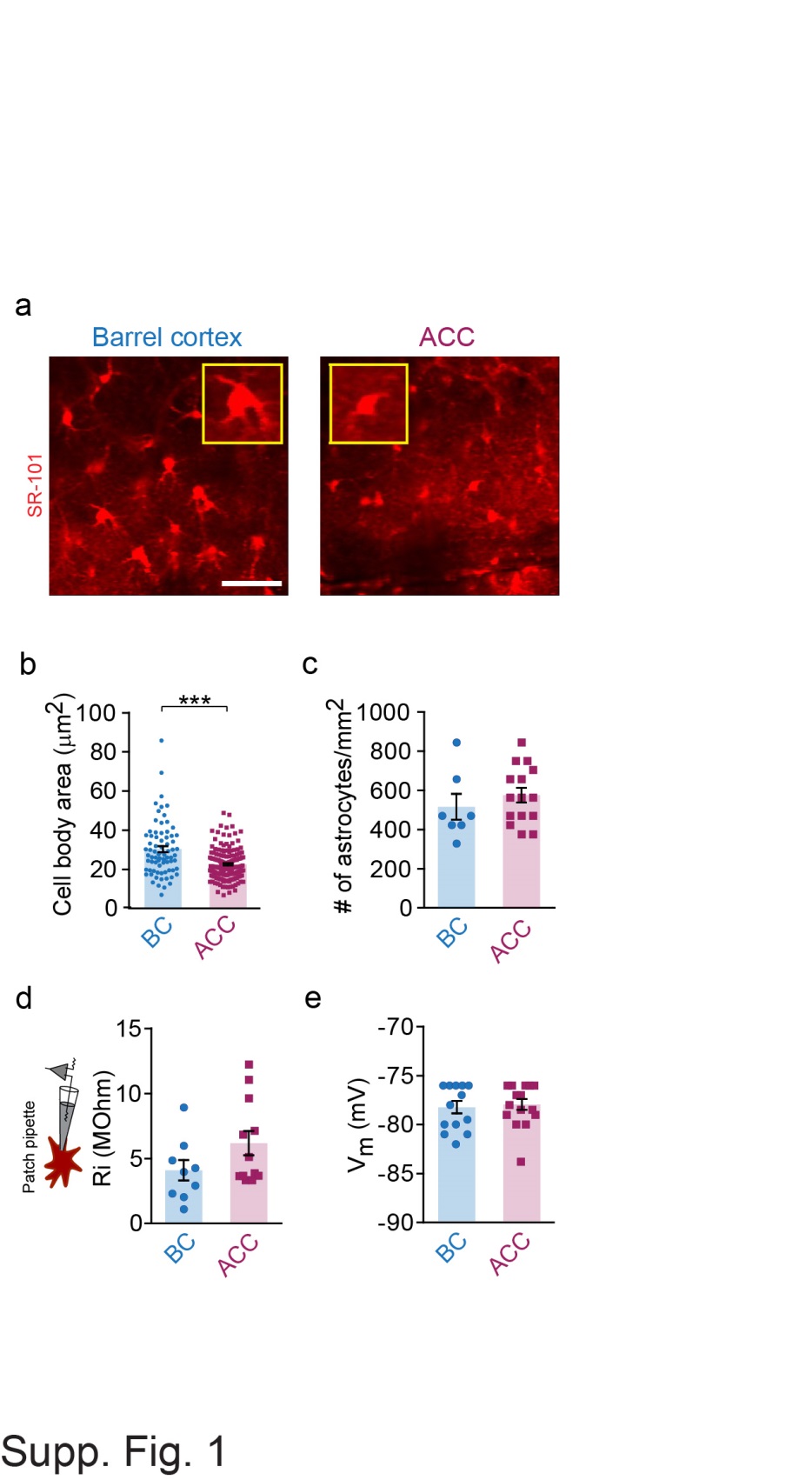
**

**Supplementary** Fig 1. Morphological and electrophysiological characteristics of astrocytes in the ACC and in the BC. a Astrocytes in layer 1 of the barrel cortex (BC) and anterior cingulate cortex (ACC) were stained with the Sulforhodamine 101 dye (SR-101) and visualized using two-photon imaging. Scale bar = 40 μm b Cell body area of astrocytes in layer 1 of the barrel cortex (29.8 ± 1.53 μm^2^, *n* = 77 cells) were significantly larger than those of the ACC (22.1 ± 0.57 μm^2^, *n* = 180 cells *P****< 0.0001). **c** There was no difference in the number of astrocytes per mm^2^ in layer 1 of the BC (516 ± 66 astrocytes/mm^2^) and ACC (575.5 ± 38 astrocytes/mm^2^ *P* = 0.41). **d** The input resistance of astrocytes in the BC (4.03 ± 0.8 MOhm, *n* = 9 cells) was slightly but not significantly smaller than that of astrocytes in the ACC (6.12 ± 0.93 MOhm, *n* = 12 cells *P* = 0.12). **e** Astrocytes in the BC have similar resting membrane potentials (V_m_ = -78.35 ± 0.64 mV, *n* = 13 cells, *N* = 7 mice) to those in the ACC (-78.05 ± 0.56 mV, *n* = 15 cells, *N* = 7 mice, *P* = 0.73). Data are mean ± SEM. Two-tailed unpaired *t* test.

**
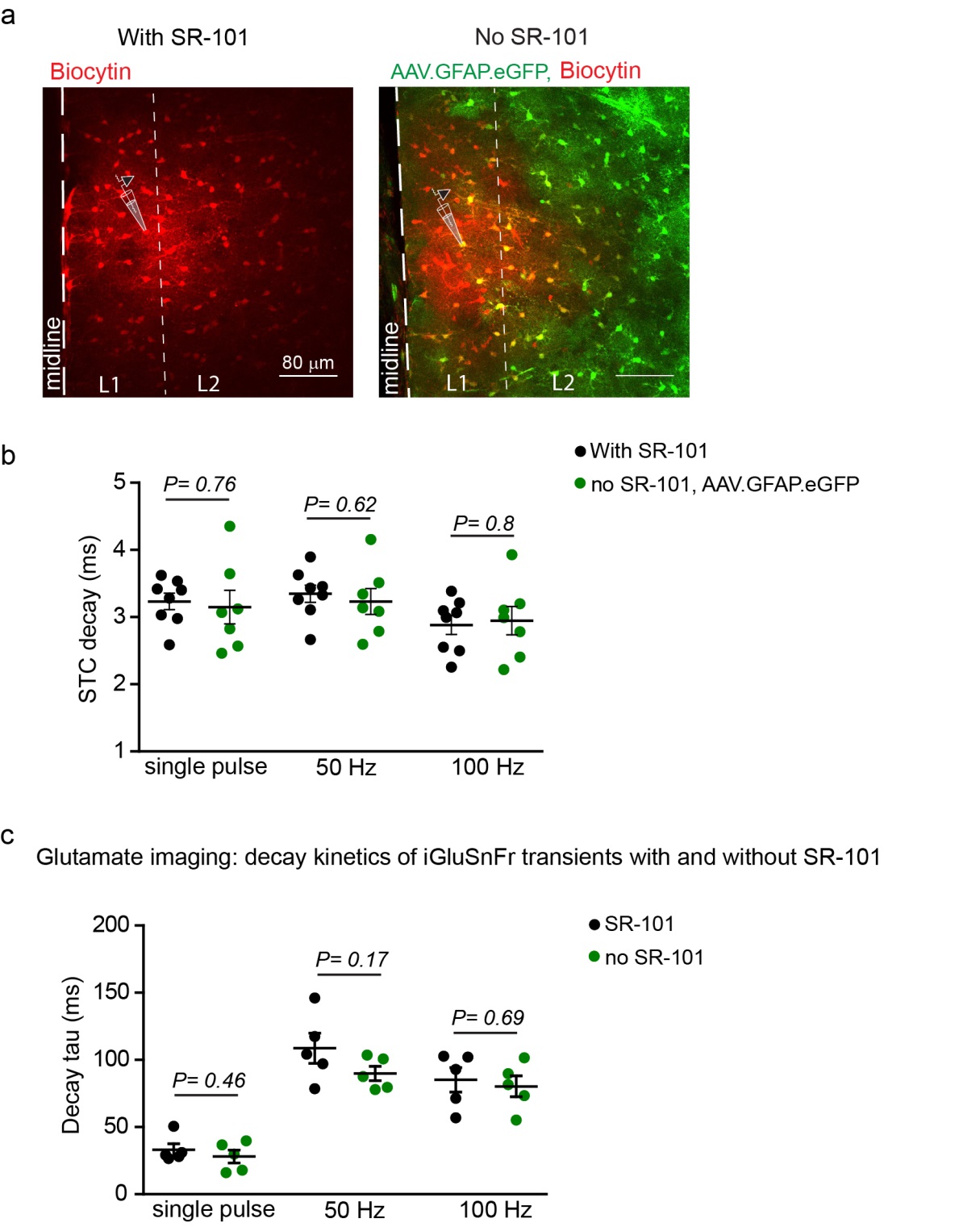
**

**Supplementary** Fig 2. Sulforhodamine-101 does not affect STC and iGluSnFr kinetics. a Biocytin staining (red) of a whole-cell patched astrocyte in L1 of ACC in a slice that was incubated in Sulforhodamine 101 (SR-101, left) and in a slice without SR-101 (Right). In slices that were not incubated with SR-101, astrocytes were recognized using the fluorescent protein EGFP that was expressed in astrocytes *via* an adeno-associated virus injected in the ACC two weeks prior to the experiment. Since biocytin diffuses through gap junctions, many astrocytes surrounding the patched one are also labelled. b The decay kinetics of glutamate transporter currents (STCs) are similar in slices with SR-101 (*n* = 8 cells, *N* = 3 mice) and without SR-101 (*n* = 7 cells, *N* = 3 mice) at all stimulation intensities. c Glutamate imaging experiments show that decay kinetics of glutamate transients are comparable in slices that were incubated with SR-101 (*n* = 5 slices) and without SR-101 (*n* = 5 slices) at all stimulation frequencies. Data are mean ± SEM. Two-way RM ANOVA test.

**
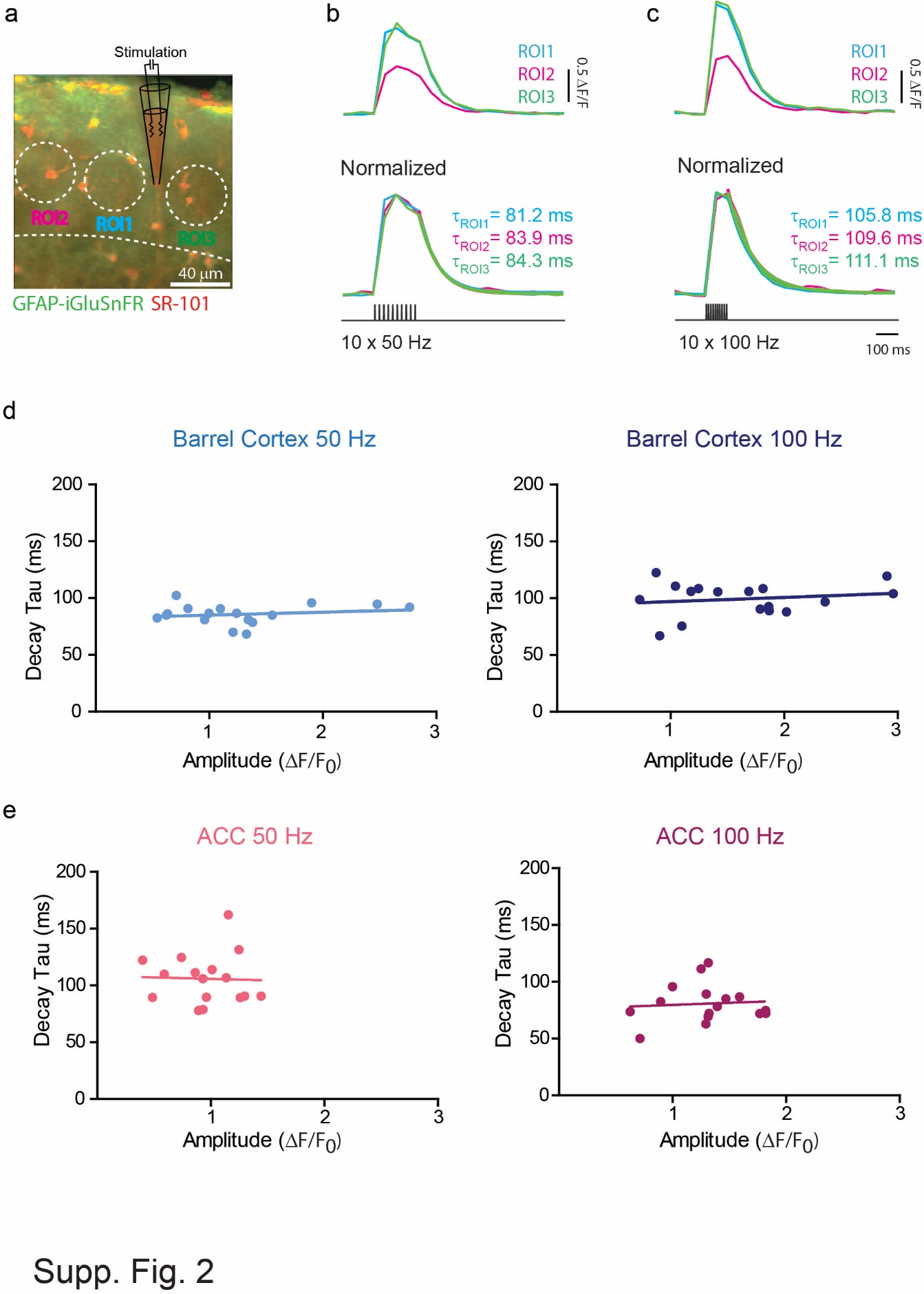
**

Supplementary Fig 3. The decay kinetics of synaptically-evoked iGluSnFr responses do not depend on the distance from the stimulation electrode nor on the magnitude of glutamate release. **a** Representative two-photon image of iGluSnFr expression (green) and SR-101 staining (red) in layer one of the barrel cortex. Circles indicate the regions of interest (ROI) at different locations relative to the tip of the theta electrode used for focal synaptic stimulation. **b** Average responses to 50 Hz synaptic stimulation detected in the 3 ROIs. Note that the amplitude of the responses depend on the distance from the stimulation electrode (upper panel) but not the decay Tau (bottom panels, traces normalized to the peak). Insets report decay kinetics for the 3 ROIs. **c** Same as **b** for 100 Hz stimulation. Note that the decay Tau upon 100 Hz stimulation is always slower than that evoked by 50 Hz stimulation, independent of the ROI location. **d** Linear regression plots showing no correlation between GFAP-iGluSnFr response size and decay Tau following focal synaptic stimulation at 50 Hz (**a**, *r^2^* = 0.035, *P* = 0.471) and 100 Hz stimulation (**b**, *r^2^* = 0.029, *P* = 0.509) in the barrel cortex. **e** Same recordings from the anterior cingulate cortex provide similar results (50 Hz: *r^2^* = 0.001, *P* = 0.897; 100 Hz: *r^2^* = 0.006, *P* = 0.774). For all frequencies, 10 pulses were applied. Data are mean ± SEM. Two-tailed unpaired *t* test.

**
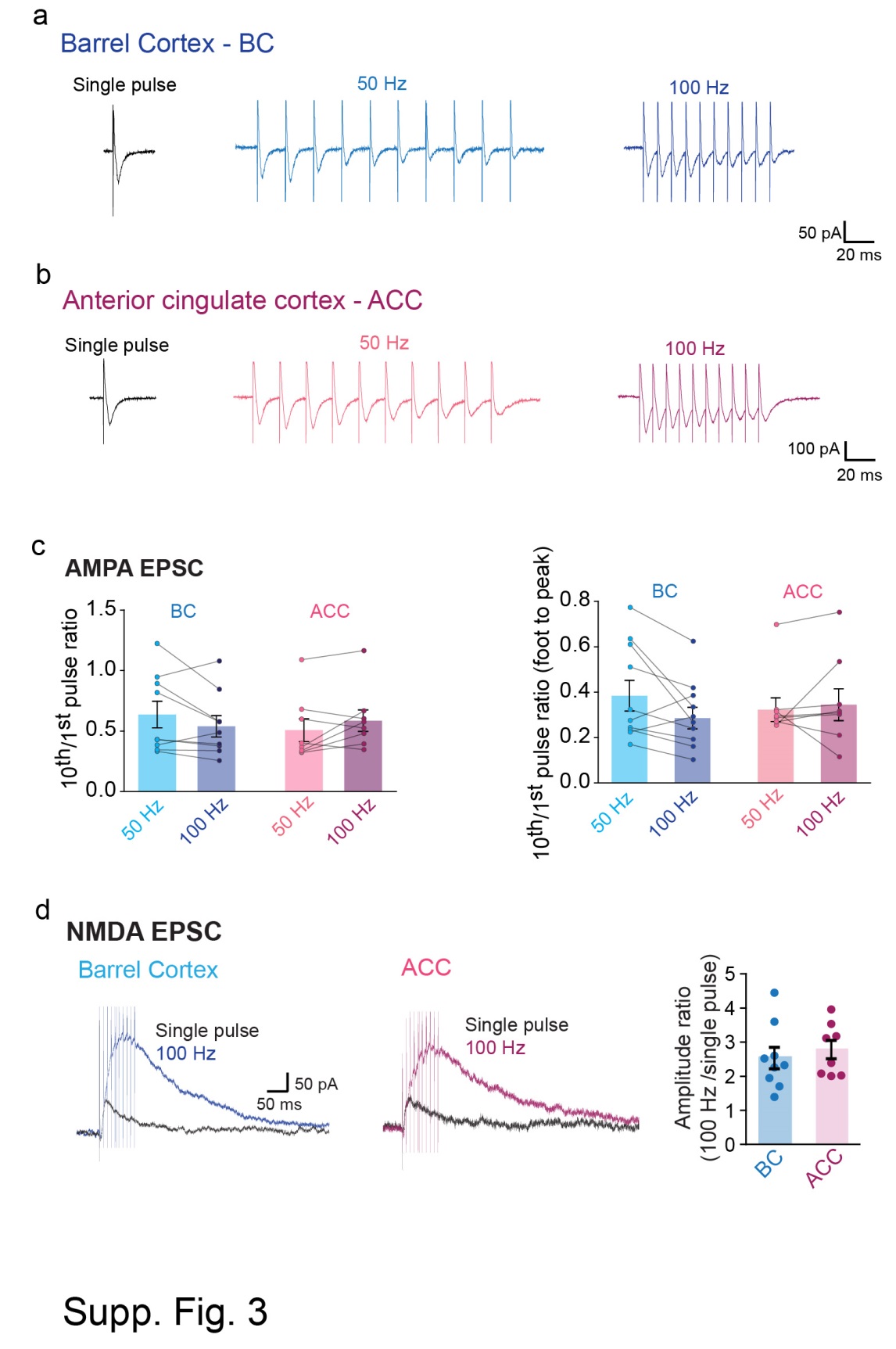
**

Supplementary **Fig 4.** Short-term synaptic plasticity in layer 5 pyramidal neurons is similar in the BC and the ACC. **a-b** Example traces of AMPA mediated EPSCs evoked by single pulse, trains of 50 Hz and trains of 100 Hz stimulations through a stimulation electrode placed in layer 1 in the BC (a) and in the ACC (**b)**. **c** *left panel.* 10^th^ to 1^st^ pulse ratio of AMPA EPSCs recorded from L5 pyramidal neurons in the BC (blue bars) at 50 Hz (0.64 ± 0.1) and at 100 Hz (0.54 ± 0.09) *n* = 9 cells, *N* = 5 mice, *P* = 0.14. Same experiments in the ACC (pink bars) at 50 Hz (0.51 ± 0.09) and at 100 Hz (0.58 ± 0.08) *n* = 8 cells, *N* = 4 mice, *P* = 0.17. *Right panel,* similar to left panel, however the 10th pulse amplitude at the peak is compared to the baseline following the last pulse not to the baseline preceding the first pulse. AMPA EPSCs 10^th^ to 1^st^ ratio in the BC at 50 Hz (0.38 ± 0.07) and at 100 Hz (0.29 ± 0.07) *n* = 9, *P* = 0.06. Same experiments in the ACC at 50 Hz (0.32 ± 0.05) and at 100 Hz (0.34 ± 0.07) *n* = 8, *P* = 0.66. **d** *Left,* Example traces of NMDA EPSCs evoked by a single pulse (black traces) and by 10 pulses of 100 Hz in the BC (blue traces) and in the ACC (pink traces). *Right,* the amplitude ratio of the last pulse of NMDA evoked EPSCs at 100 Hz over that evoked by single pulse stimulation in the BC is similar to that in the ACC (BC: 2.53 ± 0.32, *n* = 9 cells, *N* = 6 mice; ACC: 2.78 ± 0.27, *n* = 8 cells, *N* = 6 mice, *P* = 0.55). Data are mean ± SEM. Two-tailed paired *t* test and two-tailed unpaired *t* test.

**
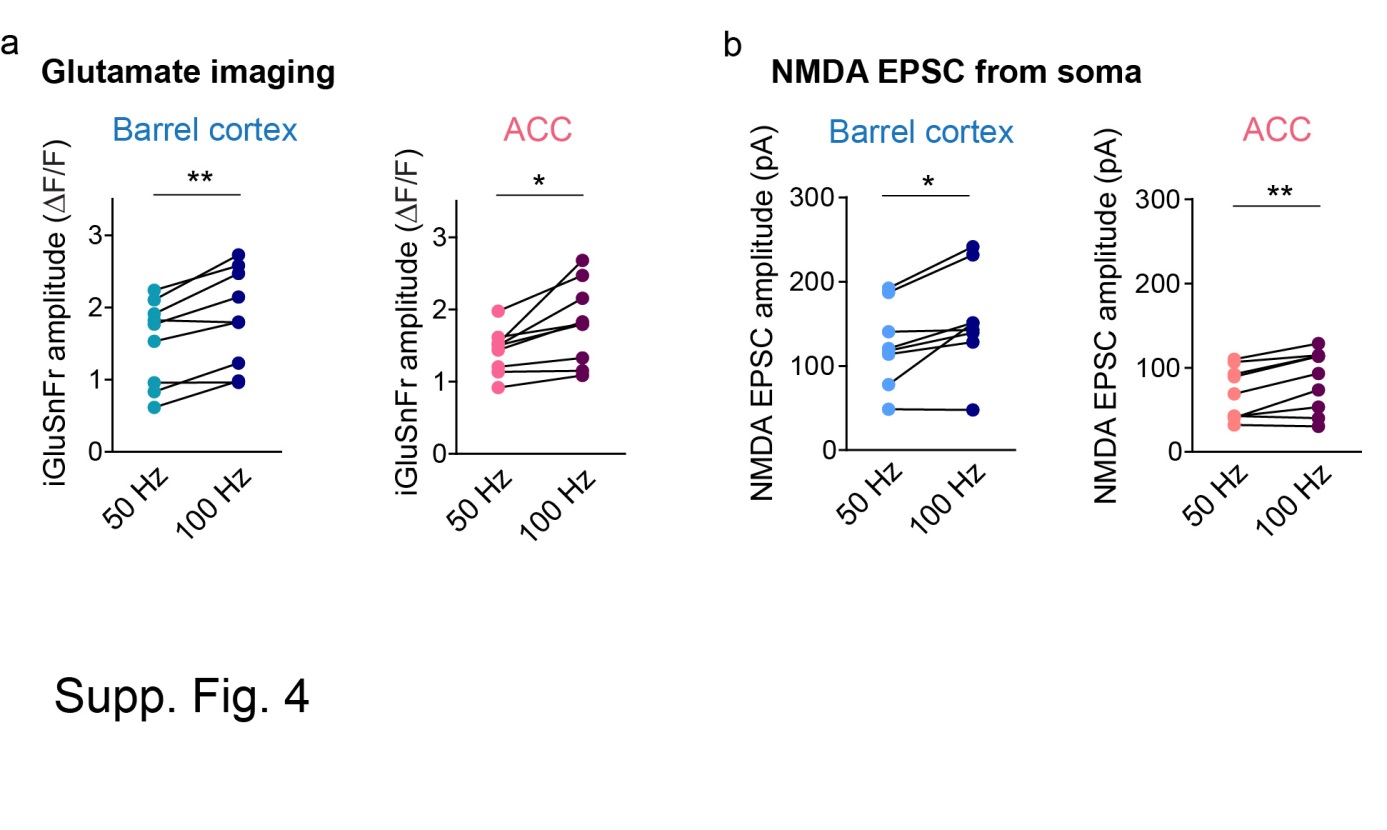
**

Supplementary **Fig 5.** Amplitude of iGluSnFr-mediated signals and NMDA-mediated EPSCs are higher at 100 Hz compared to 50 Hz in both the barrel cortex and the ACC. **a** Amplitude of iGluSnFr signals significantly increase at 100 Hz compared to 50 Hz in both the BC (blue, 50 Hz: 1.53 ± 0.2 ΔF/F, 100 Hz: 1.85 ± 0.2 ΔF/F; *n* *=* 9, *P*** = 0.002) and the ACC (pink, 50 Hz: 1.42 ± 0.1 ΔF/F, 100 Hz: 1.81 ± 0.2 ΔF/F; *n* *=* 9, *P** = 0.01). **b** Amplitude of NMDA-mediated EPSCs recorded from soma of L5 pyramidal cells significantly increase at 100 Hz compared to 50 Hz in both the BC (blue, 50 Hz: 124.8 ± 17.3 pA, 100 Hz: 153.9 ± 21.5 pA; *n* *=* 8, *P** = 0.013) and the ACC (pink, 50 Hz: 69.3 ± 10.3 pA, 100 Hz: 84.5 ± 12.2 pA; *n* *=* 9, *P*** = 0.006). Data are mean ± SEM. Two-tailed paired *t* test.

**
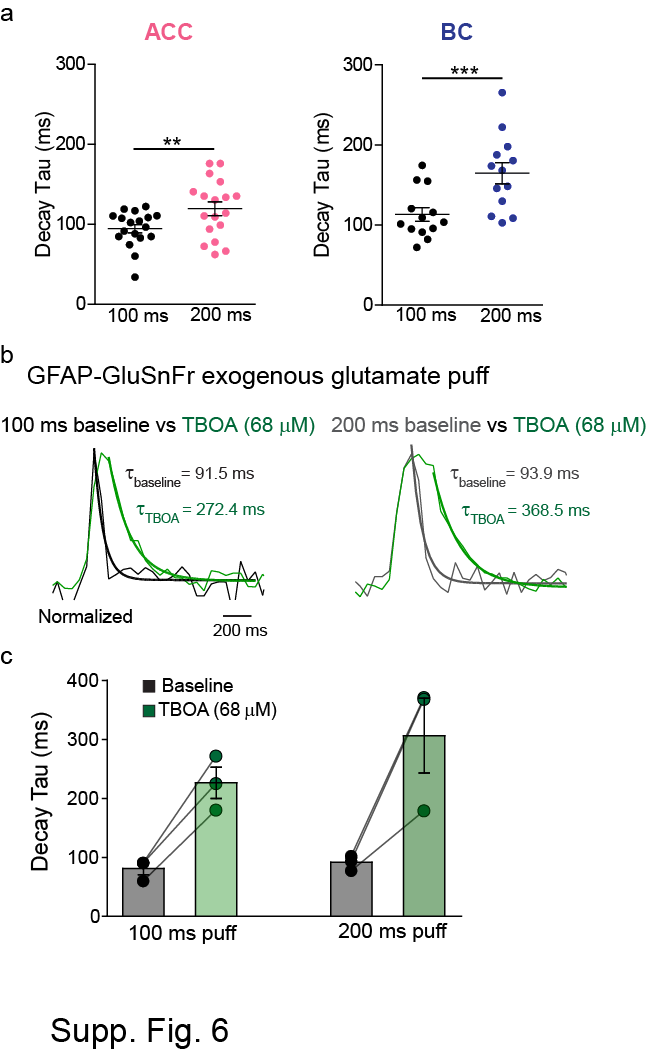
**

Supplementary **Fig 6.** The anterior cingulate cortex (ACC) copes better with high concentrations of puffed glutamate than the barrel cortex (BC). **a** In the ACC, glutamate puff-evoked iGluSnFr transients are 25% slower upon puffs of 200 ms compared to puffs of 100 ms (left panel, *n* = 18, *P***= 0.009). However, in the BC, the iGluSnFr decay kinetics are 45% slower upon glutamate puffs of 200 ms compared to puffs of 100 ms (right panel, *n* = 13, *P****= 0.0001). **b** Example traces of iGluSnFr responses evoked by continuous glutamate puffs for 100 ms (left) and for 200 ms (right) at baseline (black traces) and in the presence of DL-TBOA (68 µM, green traces) in the ACC. **c** Subsaturating concentrations of DL-TBOA strongly slowed down the decay of iGluSnFr responses for 100 ms puffs by 140% and for 200 ms puffs by 158% compared to baseline (for 100 ms puffs: baseline: 80.9 ± 10.3 ms, TBOA: 226.3 ± 26.3 ms, *n* = 3, *P** = 0.015; and for 200 ms puffs: baseline: 91.6 ± 0.2, TBOA: 306.3 ± 7.3 ms; *n* = 3 slices , *P* = 0.06, *N* = 3 mice). Traces normalized to the peak. Data are mean ± SEM.

**
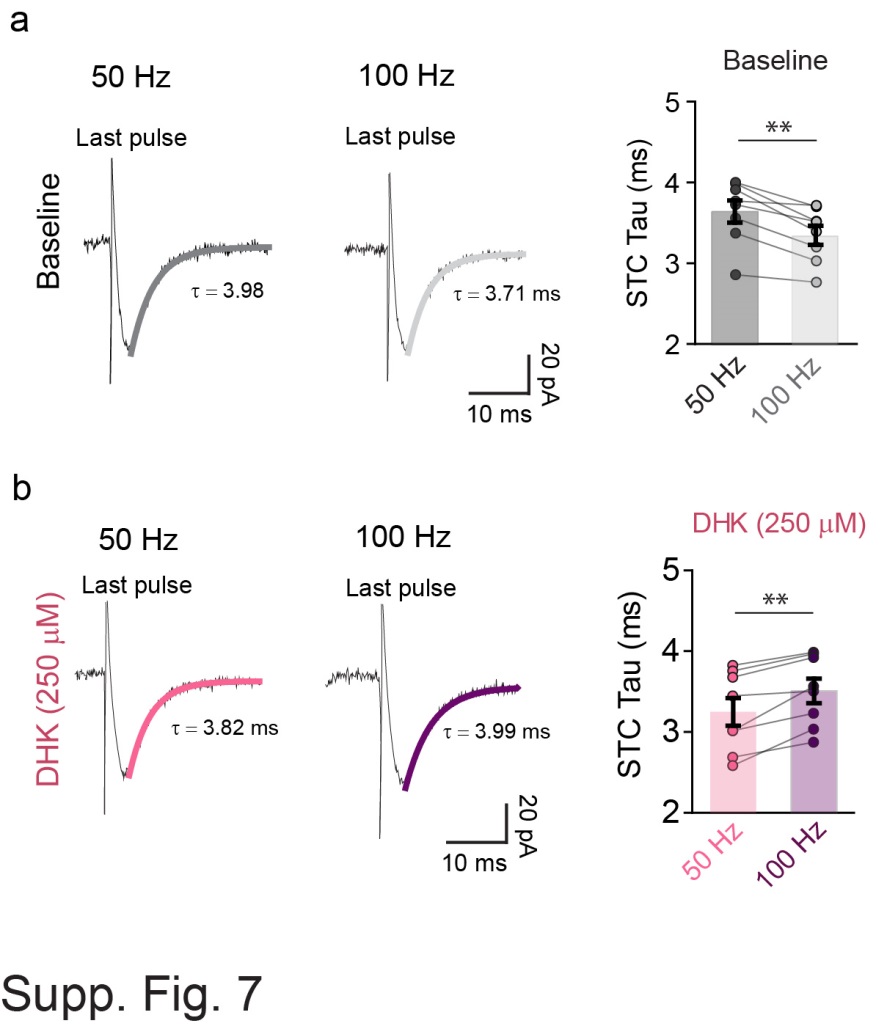
**

Supplementary Fig 7. Complete blockade of GLT-1 transporters reverses the facilitation of glutamate transporter currents at 100 Hz in the ACC. a *Left,* Representative traces of synaptically-evoked glutamate transporter currents (STC) evoked by trains of high frequency stimulations (50 Hz and 100 Hz) recorded from astrocytes in layer 1 of the ACC reporting the decay kinetics of the last pulse. *Right,* during baseline, the decay kinetics of the STC were slower at 50 Hz than at 100 Hz (Baseline, 50 Hz: 3.65 ± 0.14 ms, 100 Hz: 3.36 ± 0.12 ms; *n* = 8 cells, *P*** = 0.015). b *Left,* Same as a but in the presence of GLT-1 specific blocker DHK (250 μM) that was applied to the bath following baseline recordings. DHK significantly slowed down the decay kinetics of the STC following 100 Hz stimulations, making them slower than those evoked at 50 Hz (DHK, 50 Hz: 3.25 ± 0.17 ms, 100 Hz: 3.51 ± 0.15 ms; *n* = 8, *N* = 5 mice, *P*** = 0.026). Data are mean ± SEM. Two-tailed paired *t* test.

**
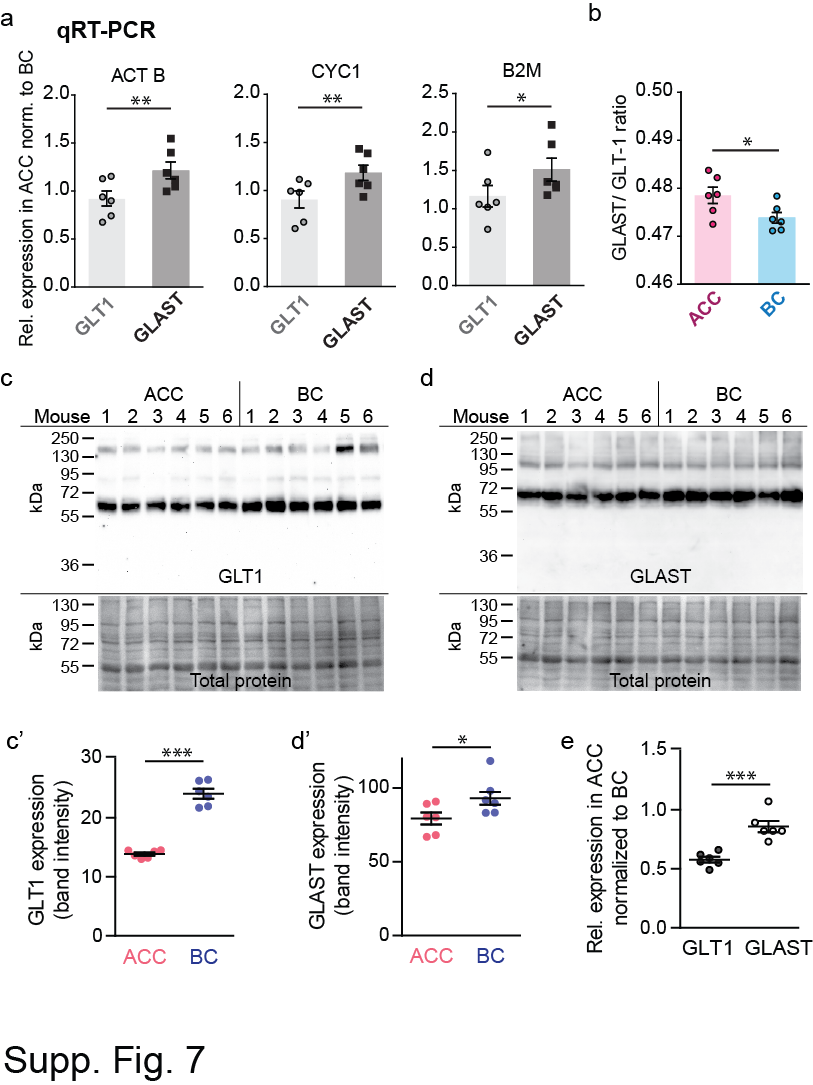
**

Supplementary **Fig 8.** Relative expression of GLT-1 and GLAST in the anterior cingulate cortex (ACC) and barrel cortex (BC). **a** qRT-PCR analysis with three different internal controls ACT B, CYC1 and B2M reveals that both GLT-1 and GLAST are expressed in the BC and ACC. The relative expression of GLAST is higher in the ACC compared to the BC (ACT B: GLT1 = 0.9 ± 0.07, GLAST = 1.2 ± 0.07, *P*** = 0.004; CYC1: GLT1 = 0.9 ± 0.08, GLAST = 1.2 ± 0.07, *P*** = 0.003; with B2M: GLT1 = 1.1 ± 0.1, GLAST = 1.5 ± 0.1, *P** = 0.01; *N* = 6 animals). **b** GLAST/GLT-1 ratio is significantly higher in the frontal cortex (ACC) compared to the BC (in ACC = 0.48 ± 0.002 and in BC = 0.47 ± 0.001; *N* = 6 mice, *P** = 0.04). Data are mean ± SEM, two-tailed paired *t* test. **c-d** Western blots for GLT1 and GLAST (upper panels) and total blotted protein used for normalization (lower panels) derived from 6 individual mice are shown. For quantification of GLT1 (**c’**) and GLAST (**d’**) band intensities were normalized to the total protein in the corresponding lanes. **e** Relative expression of GLT1 and GLAST in ACC normalized to their expression in BC. Data are the mean ± SEM. Two-tailed unpaired *t* test; *N* = 6 mice, **P* < 0.046, ****P* < 0.0004, *****P* < 0.0001.

**
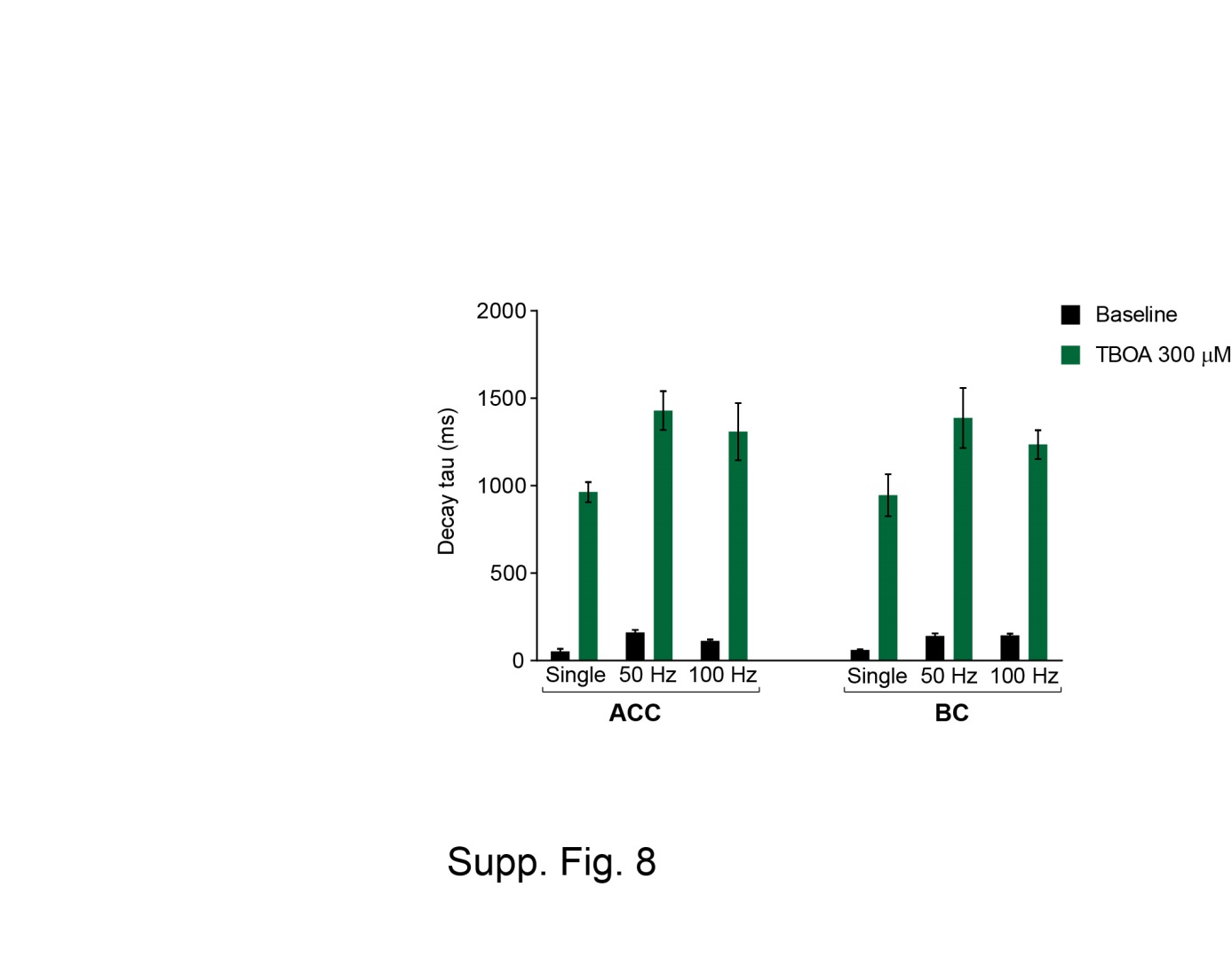
**

Supplementary **Fig 9.** Full blockade of both transporters with 300 µM DL-TBOA leads to similar synaptically-evoked iGluSnFR signals in the ACC and BC. In ACC: Single pulse: Baseline 52.8 ± 14.4 ms, TBOA_300_ 963.4 ± 57 ms; 50 Hz: Baseline 160.4 ± 15.4 ms, TBOA_300_ 1430 ± 110.2 ms; 100 Hz: Baseline 112.7 ± 8.5 ms, TBOA_300_ 1309.5 ± 163 ms; *n* = 4 slices, *N* = 2 mice, *P_single_***<0.01, *P_50Hz, 100Hz_****<0.0001. In BC: Single pulse: Baseline 61.1 ± 3.5 ms, TBOA_300_ 945.3 ± 120 ms; 50 Hz: Baseline 141.6 ± 13.4 ms, TBOA_300_ 1387.4 ± 171.2 ms; 100 Hz: Baseline 144.7 ± 9.7 ms, TBOA_300_ 1235.1 ± 82.4 ms; *n* = 4 slices, *N* = 2 mice, *P_single_****<0.001, *P_50Hz, 100Hz_****<0.0001. Data are mean ± SEM. Two-tailed paired *t* test. 2-way RM ANOVA test. Please note that in a number of recordings, upon 300 µM TBOA application, we observed a change in baseline iGluSnFr fluorescence, a reduced amplitude of the evoked responses and cellular swelling often accompanied by a lateral or Z drift. These experiments had to be excluded, which explains the low *n* number for this experimental set.

Supplementary Table 1. Forward and reverse sequences of primers used in the qRT-PCR experiment

| *ActB* F | MusACTB F | CTTCCTCCCTGGAGAAGAGC |
| --- | --- | --- |
| *ActB* R | MusACTB R | ATGCCACAGGATTCCATACC |
| *Cyc1* F | MusCYC1 F | TGCTACACGGAGGAAGAAGC |
| *Cyc1* R | MusCYC1 R | CCATCATCATTAGGGCCATC |
| *Slc1a2* F | MusSLC1A2 F1098 | GATGCCTTCCTGGATCTCATT |
| *Slc1a2* R | MusSLC1A2 R1182 | TCTTTGTCACTGTCTGAATCTGC |
| *Slc1a3* F | MusSLC1A3 F1412 | CGCGGTGATAATGTGGTATGC |
| *Slc1a3* R | MusSLC1A3 R1509 | CAAGCTGTCCCCCAATCACA |
| *B2m* F | MusB2M F34 | GTCGCTTCAGTCGTCAGCAT |
| *B2m* R | MusB2M R128 | TGAGGGGTTTTCTGGATAGCAT |

Primers for the internal controls ACT B (*ActB* F, *ActB* R), CYC1 (*Cyc1* F, *Cyc1* R), B2M (*B2m* F, *B2m* R) and the primers for the genes encoding GLT-1 (*Slc1a2* F, *Slc1a2* R) and GLAST (*Slc1a3* F, *Slc1a3* R).

**Supplementary Discussion**

We measured the relative time course of extrasynaptic glutamate and glutamate uptake with two different techniques: STCs recorded from individual astrocytes and glutamate-induced iGluSnFr fluorescence intensity changes with two-photon microscopy. Although the decay time constants of signals appear to be sensitive to minor changes in glutamate uptake [^1-4^](#_ENREF_1), these measures have to be interpreted with caution, as they most likely do not reflect the real glutamate time course in the extrasynaptic space. Hence, these measures are inevitably affected by a number of factors that depend on the detection methods. STC kinetics are distorted by the filtering properties of the astrocyte membrane that slow down the decay of the currents [^5^](#_ENREF_5). Additionally, STC may also contain non-GluTs mediated components. iGluSnFr glutamate imaging, as other imaging methods (such as those based on GCaMPs), is instead influenced by the biophysical properties of the sensors and the kinetics of the indicator that are ultimately limited by the structural change that reconstitutes the fluorescent complex. Notably, iGluSnFR shows a Ƭ_off_ for synaptically released glutamate of 13.8 ms, i.e. substantially slower than the estimated time course of glutamate in the extrasynaptic space [^6^](#_ENREF_6). Yet, since the membrane-bound form of iGluSnFr possesses a high affinity for glutamate (3.1 µM), it appears to represent a suitable sensor to measure physiologically relevant glutamate concentrations (notice that NMDAR affinity is ̴2 µM) [^7^](#_ENREF_7)^,^[^8^](#_ENREF_8). Hence, the high affinity for glutamate of the iGluSnFr used in the present study, the independency of its decay to short-term synaptic plasticity (Fig. 3, Supplementary Fig. 4) and its high sensitivity to changes in glutamate clearance and lifetime [^1-4^](#_ENREF_1), render this imaging method an adequate tool to study relative variations in brain glutamate uptake. Moreover, it is important to note that we measure changes in extrasynaptic glutamate, which reach much lower concentrations than synaptic glutamate, the latter being more difficult to measure because of possible saturation of the sensor close to the synaptic release site. We are nevertheless confident that the recorded iGluSnFr signals were far from saturation because glutamate transporter blockers consistently increased the amplitude of the signals even at 100 Hz, i.e. when the initial response was maximal (baseline 1.71 ± 0.09 dF/F, DHK 3.330 ± 0.17 dF/F, n = 32, P < 0.00001).

**Supplementary References**

1. Armbruster, M., Hanson, E. & Dulla, C.G. Glutamate Clearance Is Locally Modulated by Presynaptic Neuronal Activity in the Cerebral Cortex. *J Neurosci* **36**, 10404-10415 (2016).

2. Parsons, M.P. *et al.* Real-time imaging of glutamate clearance reveals normal striatal uptake in Huntington disease mouse models. *Nat Commun* **7**, 11251 (2016).

3. Jiang, R., Diaz-Castro, B., Looger, L.L. & Khakh, B.S. Dysfunctional Calcium and Glutamate Signaling in Striatal Astrocytes from Huntington's Disease Model Mice. *J Neurosci* **36**, 3453-3470 (2016).

4. Capuani, C. *et al.* Defective glutamate and K+ clearance by cortical astrocytes in familial hemiplegic migraine type 2. *EMBO Mol Med* **8**, 967-986 (2016).

5. Diamond, J.S. Deriving the glutamate clearance time course from transporter currents in CA1 hippocampal astrocytes: transmitter uptake gets faster during development. *J Neurosci* **25**, 2906-2916 (2005).

6. Clements, J.D., Lester, R.A., Tong, G., Jahr, C.E. & Westbrook, G.L. The time course of glutamate in the synaptic cleft. *Science* **258**, 1498-1501 (1992).

7. Hanson, E. *et al.* Astrocytic glutamate uptake is slow and does not limit neuronal NMDA receptor activation in the neonatal neocortex. *Glia* **63**, 1784-1796 (2015).

8. Herman, M.A. & Jahr, C.E. Extracellular glutamate concentration in hippocampal slice. *J Neurosci* **27**, 9736-9741 (2007).
