# Supplementary material for: Differences in glutamate uptake between cortical regions impact neuronal NMDA receptor activation
Source: Commun Biol. 2019 Apr 5;2:127. doi: 10.1038/s42003-019-0367-9 (PMC6451009; doi:10.1038/s42003-019-0367-9)
Supplement: Supplementary file 2 — Reporting Summary [file 42003_2019_367_MOESM2_ESM.pdf]

nature research

Corresponding author(s): Mirko Santello

Last updated by author(s): Mirko Santello, Jennifer Romanos

Reporting Summary

Nature Research wishes to improve the reproducibility of the work that we publish. This form provides structure for consistency and transparency in reporting. For further information on Nature Research policies, see Authors & Referees and the Editorial Policy Checklist.

Statistics

For all statistical analyses, confirm that the following items are present in the figure legend, table legend, main text, or Methods section.

n/a	Confirmed	
☐	☒	The exact sample size (*n*) for each experimental group/condition, given as a discrete number and unit of measurement
☐	☒	A statement on whether measurements were taken from distinct samples or whether the same sample was measured repeatedly
☐	☒	The statistical test(s) used AND whether they are one- or two-sided<br>*Only common tests should be described solely by name; describe more complex techniques in the Methods section.*
☐	☒	A description of all covariates tested
☐	☒	A description of any assumptions or corrections, such as tests of normality and adjustment for multiple comparisons
☐	☒	A full description of the statistical parameters including central tendency (e.g. means) or other basic estimates (e.g. regression coefficient) AND variation (e.g. standard deviation) or associated estimates of uncertainty (e.g. confidence intervals)
☐	☒	For null hypothesis testing, the test statistic (e.g. *F*, *t*, *r*) with confidence intervals, effect sizes, degrees of freedom and *P* value noted<br>*Give P values as exact values whenever suitable.*
☒	☐	For Bayesian analysis, information on the choice of priors and Markov chain Monte Carlo settings
☒	☐	For hierarchical and complex designs, identification of the appropriate level for tests and full reporting of outcomes
☒	☐	Estimates of effect sizes (e.g. Cohen's *d*, Pearson's *r*), indicating how they were calculated

Our web collection on statistics for biologists contains articles on many of the points above.

Software and code

Policy information about availability of computer code

Data collection	n/A
Data analysis	n/A

For manuscripts utilizing custom algorithms or software that are central to the research but not yet described in published literature, software must be made available to editors/reviewers. We strongly encourage code deposition in a community repository (e.g. GitHub). See the Nature Research guidelines for submitting code & software for further information.

Data

Policy information about availability of data

All manuscripts must include a data availability statement. This statement should provide the following information, where applicable:
- Accession codes, unique identifiers, or web links for publicly available datasets
- A list of figures that have associated raw data
- A description of any restrictions on data availability

Data, associated protocols and additional information regarding this paper are available to the reader upon request from the authors and are located on the internal server of the institute of Pharmacology and Toxicology at the University of Zurich.

Field-specific reporting

Please select the one below that is the best fit for your research. If you are not sure, read the appropriate sections before making your selection.

☒ Life sciences ☐ Behavioural & social sciences ☐ Ecological, evolutionary & environmental sciences

October 2018

Life sciences study design

All studies must disclose on these points even when the disclosure is negative.

Sample size	Our sample size is similar to that reported in previous publications and is appropriate for our statistical tests.
Data exclusions	In a number of recordings in which we applied 300 µM DL-TBOA, we observed a change in baseline iGluSnFr fluorescence, a reduced amplitude of the evoked responses and cellular swelling often accompanied by a lateral or Z drift. These experiments had to be excluded.<br>STC recordings: recordings with an access resistance changing more than 30% between the beginning and the end of the recording were discarded.<br>Synaptic current recordings: recordings with an access resistance > 15 MΩ were discarded.
Replication	All experiments were repeated several times in several animals (see our n and N values)
Randomization	Both male and female mice were used for experiments and randomly assigned to experimental groups as stated in the methods section.
Blinding	In general the electrophysiological and functional imaging experiments in brain slices are not performed blind, since they involve measurements of cellular currents/transients with minimal possibility of bias of the results by the experimenter. Also the analysis (mainly consisting in best fitting current decays with an exponential function in this study) is much less subject to possible bias than that of the immunocytochemical studies and is usually not performed blind.<br>In most of the experiments we only used one animal group (C57BL6 mice) so no blinding was done.<br>In one set of experiments we used functional GLAST KO mice blinding was done.

Reporting for specific materials, systems and methods

We require information from authors about some types of materials, experimental systems and methods used in many studies. Here, indicate whether each material, system or method listed is relevant to your study. If you are not sure if a list item applies to your research, read the appropriate section before selecting a response.

Materials & experimental systems

n/a	Involved in the study	
☐	☒	Antibodies
☒	☐	Eukaryotic cell lines
☒	☐	Palaeontology
☐	☒	Animals and other organisms
☒	☐	Human research participants
☒	☐	Clinical data

Methods

n/a	Involved in the study	
☒	☐	ChIP-seq
☒	☐	Flow cytometry
☒	☐	MRI-based neuroimaging

Antibodies

Antibodies used	Alexa 647-streptavidin, anti-GLAST and anti-GLT1 antibodies.
Validation	anti-GLT1 antibodies (1:700, rabbit polyclonal, knock-out verified, Synaptic Systems, Cat. No. 250203)<br>anti-GLAST antibodies (1:1500, rabbit polyclonal, knock-out verified, Synaptic Systems, Cat. No. 250113)<br>Alexa 647-streptavidin (1:700, Jackson ImmunoResearch Europe Ltd, code: 016-600-084)

Animals and other organisms

Policy information about studies involving animals; ARRIVE guidelines recommended for reporting animal research

Laboratory animals	Permission for animal experiments was obtained from the Tierversuchskommission of the canton of Zurich, Zurich, Switzerland.<br>Adult 5 to 8 week-old C57BL/6J male and female mice were group housed in filtered cages with a standard 12-hour light/dark cycle and food and water available ad libitum. Mice were housed with a maximum of 5 animals per cage.<br>Homozygous GLAST-CreERT2 knock-in mice have been used as functional GLAST-KO mice.
Wild animals	n/A
Field-collected samples	n/A
Ethics oversight	n/A

Note that full information on the approval of the study protocol must also be provided in the manuscript.
